# Mechanistic characterization of a copper containing thiosemicarbazone with potent antitumor activity

**DOI:** 10.18632/oncotarget.16324

**Published:** 2017-03-17

**Authors:** Henning Karlsson, Mårten Fryknäs, Sara Strese, Joachim Gullbo, Gunnar Westman, Ulf Bremberg, Tobias Sjöblom, Tatjana Pandzic, Rolf Larsson, Peter Nygren

**Affiliations:** ^1^ Department of Medical Sciences, Uppsala University, Uppsala, Sweden; ^2^ Department of Immunology, Genetics and Pathology, Uppsala University, Uppsala, Sweden; ^3^ Department of Chemistry and Chemical Engineering, Chalmers University of Technology, Gothenburg, Sweden; ^4^ Department of Medicinal Chemistry, Uppsala University, Uppsala, Sweden

**Keywords:** cancer drug, thiosemicarbazone, spheroid, VLX60, BRAF

## Abstract

**Background:**

The thiosemicarbazone CD 02750 (VLX50) was recently reported as a hit compound in a phenotype-based drug screen in primary cultures of patient tumor cells. We synthesized a copper complex of VLX50, denoted VLX60, and characterized its antitumor and mechanistic properties.

**Materials and Methods:**

The cytotoxic effects and mechanistic properties of VLX60 were investigated in monolayer cultures of multiple human cell lines, in tumor cells from patients, in a 3-D spheroid cell culture system and *in vivo* and were compared with those of VLX50.

**Results:**

VLX60 showed ≥ 3-fold higher cytotoxic activity than VLX50 in 2-D cultures and, in contrast to VLX50, retained its activity in the presence of additional iron. VLX60 was effective against non-proliferative spheroids and against tumor xenografts *in vivo* in a murine model. In contrast to VLX50, gene expression analysis demonstrated that genes associated with oxidative stress were considerably enriched in cells exposed to VLX60 as was induction of reactive oxygen. VLX60 compromised the ubiquitin-proteasome system and was more active in *BRAF* mutated versus *BRAF* wild-type colon cancer cells.

**Conclusions:**

The cytotoxic effects of the copper thiosemicarbazone VLX60 differ from those of VLX50 and shows interesting features as a potential antitumor drug, notably against *BRAF* mutated colorectal cancer.

## INTRODUCTION

Thiosemicarbazones have been explored as antitumor agents since several decades and a number of compounds in this drug family have shown promising antitumor activity both *in vitro* and *in vivo* [1–8]. Triapine (3-aminopyridine-2-carboxaldehyde thiosemicarbazone) is the most comprehensively studied anticancer thiosemicarbazone and has been described as a potent inhibitor of iron containing enzymes such as ribonucleotide reductase (RR) and p53R2 [8–10]. The inhibitory effect of triapine was previously thought to be due to the direct removal of Fe from the enzymes. However, more recent data show that redox effects of iron complexes of thiosemicarbazones on these enzymes and anticancer effects through targeting of a number of other molecules, including NDRG1 and top2α, might also be important [7, 8, 11, 12]. Triapine and another novel thiosemicarbazone, DpC (Dp4cycH4mT), are currently in phase I and II clinical trials [13–15] (https://clinicaltrials.gov/ct2/show/NCT02688101) and other thiosemicarbazones, *e.g*. Dp44mT and Bp44mT, have shown potent antitumor activity in tumor xenografts in mice [16]. However, clinical trials with triapine demonstrated poor activity and side effects such as myelosuppression, hypoxia and methemoglobinemia [13–15]. An altered design of the ligand has been proposed as a way forward for this class of anticancer drugs [15].

It is well known that the formation of copper complexes of mono- and bis-thiosemicarbazones has been associated with several fold increased antitumor activity *in vitro* and *in vivo* [8, 17–22] and it was shown already in the 1960s that a powerful antitumor bis-thiosemicarbazone required nutrient copper for its activity in a rodent model [21, 23]. The success of the platinum anticancer drugs has stimulated research on metal-based drugs and the fact that a number of copper complexes have shown a broad spectrum of antitumor activities has fueled the interest to develop copper complexes as anticancer agents [18, 22, 24, 25]. Interestingly, copper complexes have also been suggested to be able to overcome platinum resistance [17, 18, 22, 24, 26]. However, little is known about their mechanisms of action and most investigations focus on the interaction with DNA [22]. Early studies with copper chelates of thiosemicarbazones indicated the ability of these compounds to induce cell death associated with generation of reactive oxygen species (ROS) and depletion of cellular glutathione [17, 19], but few papers report on the effects on intracellular signal transduction [22]. To the best of our knowledge no copper-thiosemicarbazone complex has thus far entered clinical trials. However, a phase I clinical trial (https://clinicaltrials.gov/ct2/show/NCT00742911) of a copper mixture based on co-administration of copper gluconate and disulfiram for the treatment of refractory solid tumors was recently completed and at least two other phase I-II studies, utilizing this copper combination, are planned in glioblastoma but not yet recruiting (https://clinicaltrials.gov/ct2/show/NCT01777919 and https://clinicaltrials.gov/ct2/show/NCT02715609).

**Figure 1:**
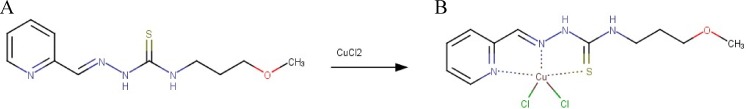
Suggested structural formulae of (**A**) VLX50 and (**B**) VLX60

We recently reported on the identification of the thiosemicarbazone 3-(3-methoxypropyl)-1-[[(pyridin-2-yl)methylidene]amino]thiourea (CD 02750, subsequently denoted VLX50) (Figure 1A) as a hit in a phenotype-based drug screen and found it to be active against ovarian carcinoma cells both *in vitro* and *in vivo* [5]. Confirmed by a series of experiments this drug was shown to deplete intracellular iron, leading to hypoxia signaling. In the present study, our aim was to develop VLX50 and rationally design a more potent drug with enhanced anticancer activity and explore its mechanism of action. Therefore, we synthesized a copper complex (Copper(II) chloride complex of 3-(3-methoxypropyl)-1-[[(pyridin-2-yl)methylidene]amino]thiourea) of VLX50 (the copper complex subsequently denoted VLX60; Figure 1B) and investigated its antitumor and mechanistic properties in various models, including xenografts in mice.

Since in the initial experiments VLX60 was found most active against a cell line from colon cancer we included colon cancer models able to associate the activity to the *KRAS* and *BRAF* mutation status, established to have predictive and/or prognostic importance in this tumor type [27, 28]. Mechanistic properties were explored using gene expression analysis of drug exposed tumor cells. Since proteasome inhibition has emerged as a putative target for copper complexes we also evaluated the effect of VLX60 on the ubiquitin-proteasome system (UPS) [22, 29–32]. Important general features of cytotoxic drugs such as effects on cell proliferation, cell cycle, and apoptosis were assessed.

## RESULTS

### Drug activity in monolayer cultured cell lines

**Figure 2:**
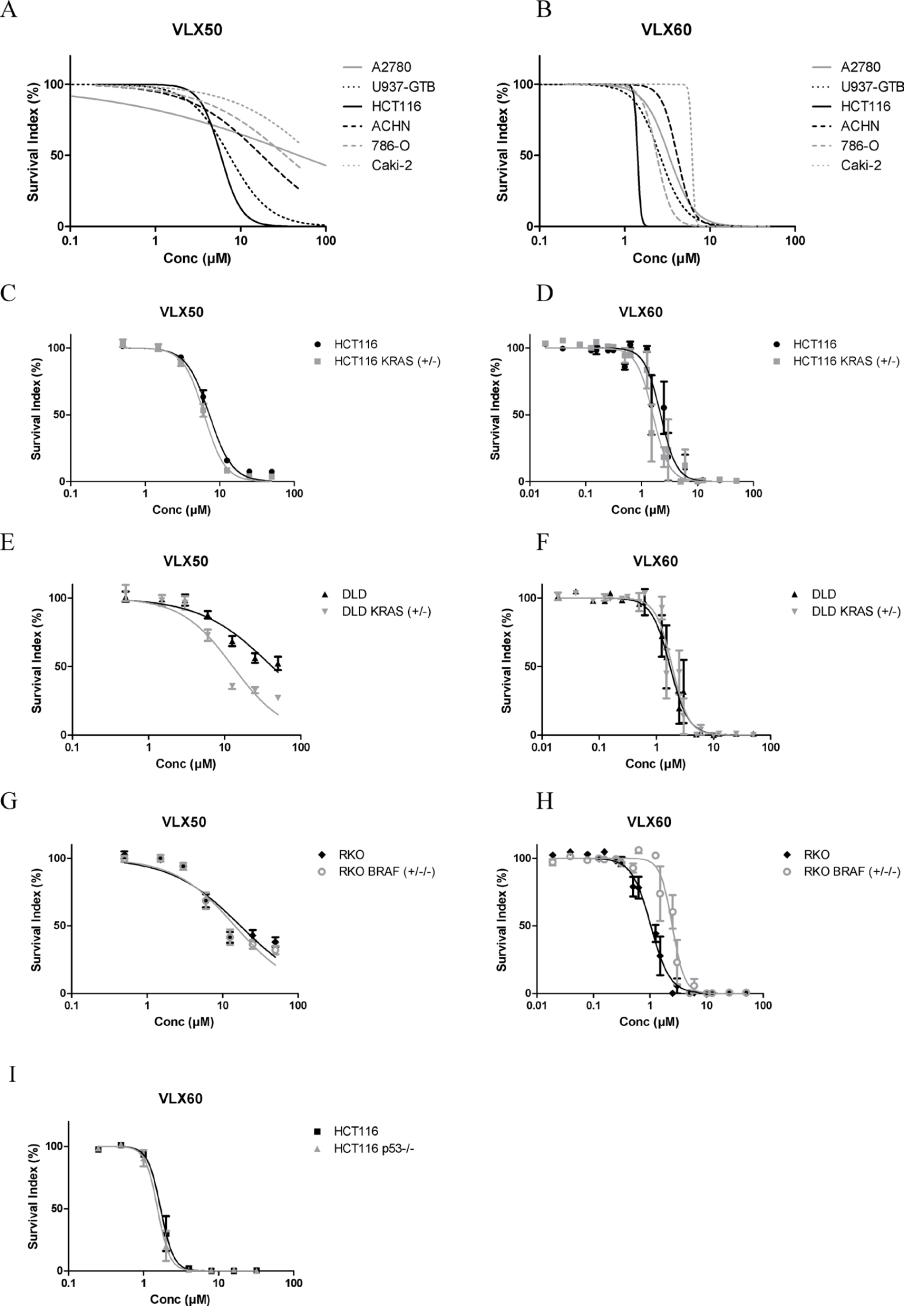
(**A**–**B**) Cell survival in the FMCA assay, expressed as survival index of cell lines cultured as monolayers when exposed to VLX50 or VLX60 for 72 h. Concentration-response curves are based on two to six independent experiments with duplicate or triplicate wells for each concentration. Standard error bars are omitted for clarity. (**C**–**H**) Antitumor activity of VLX50 and VLX60 in three isogenic cell models of colon cancer. The parental cells harbor *KRAS* or *BRAF* mutations that are knocked out in the *KRAS*/*BRAF* sublines. Concentration-response curves are based on four to eight independent experiments, with duplicate or quadruplicate wells for each drug concentration. IC_50_-values are shown in Table 1. (**I**) Nearly identical survival curves irrespective of knockout of *p53* in HCT116 cells. Concentration-response curves (mean ± SEM) are based on two independent experiments with quadruplicate wells for each concentration.

The cytotoxic effect of VLX50 and VLX60 was investigated in 6 different cancer cell lines of various origins (Figure 2A–2B). All cell lines were more sensitive to VLX60 than VLX50. The three kidney cancer cell lines ACHN, Caki-2 and 786-O as well as the ovarian cancer cell line A2780 were highly resistant to VLX50. In contrast, all cell lines showed steep drops in cell viability well below 10 μM VLX60. For both drugs, the colon cancer cell line HCT116 was the most sensitive cell line.

Given the high activity of VLX50 and VLX60 against the colon cancer cell line HCT116, which is *KRAS* mutated, the activity of these drugs was compared in three pairs of colon cancer cell lines with different *KRAS* and *BRAF* mutation status (Figure 2C–2H and Table 1). The parental cells harbor *KRAS* or *BRAF* mutations that are knocked out (KO) in the *KRAS*/*BRAF* sublines. The activity of VLX50 was significantly higher in DLD cells with wt status of *KRAS* (DLD *KRAS* (+/−) compared to cells with one mutant allele of the gene (DLD). However, *KRAS* mutation status in HCT116 cells as well as *BRAF* mutation status in RKO cells did not affect the response to VLX50. For VLX60 the activity was essentially unaffected by *KRAS* status whereas, interestingly, the RKO cells with mutant *BRAF* were statistically significantly more than 2-fold more sensitive than the RKO cells with KO of mutant *BRAF*. All six cell lines were more sensitive to VLX60 compared with VLX50. IC_50_ for VLX50 and VLX60 were 7.3 μM and 2.3 μM, respectively, in the parental HCT116 cells (Figure 2C–2D and Table 1).

**Table 1:** Antitumor activity of VLX50 and VLX60 in three isogenic cell models of colon cancer

	VLX50			VLX60		
Cell line	IC50 (μM)		95% CI	IC50 (μM)		95% CI
HCT116	7.270		5.890, 8.650	2.328		1.265, 3.390
HCT116 *KRAS* (+/−)	6.186		5.019, 7.352	2.048		0.6730, 3.424
DLD	43.83	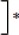	19.57, 68.09	1.965		0.9421, 2.988
DLD *KRAS* (+/−)	12.80		8.729, 16.87	1.963		1.256, 2.671
RKO	18.73		4.980, 32.47	1.015	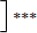	0.8345, 1.196
RKO *BRAF* (+/−/−)	15.06		6.519, 23.60	2.449		1.734, 3.163

The parental cells harbor *KRAS* or *BRAF* mutations that are knocked out in the *KRAS*/*BRAF* sublines. Results are expressed as IC_50_ ± 95% Confidence Interval (CI) and are based on four to eight independent experiments, with duplicate or quadruplicate wells for each drug concentration. When statistically significant different IC_50_-values between the parental cell line and its subline are present, it is indicated with an asterisk. *P ≤ 0.05; ***P ≤ 0.001. Concentration-response curves are shown in Figure 2C–2H.

In serum free medium, the relative difference in efficacy between the drugs remained essentially unchanged, indicating that their different potency is not due to various protein binding. However, the IC_50_ concentrations were approximately 2.5 fold lower than in the serum containing standard medium (not shown).

The activity of VLX60 was not obviously associated with *p53* status, as shown by the nearly identical survival curves irrespective of KO of *p53* in HCT116 cells (Figure 2I).

The effect of VLX50 and VLX60 was tested against a cell line derived from normal colon cells and peripheral blood mononuclear cells and compared with the effect against HCT116 cells. For both drugs, the colon cancer cell line HCT116 was more sensitive than cells from the normal cell line, but VLX60 was more active against the normal mononuclear cells compared with the tumor cells, which was not observed with VLX50 (Online Resource, Supplementary Figure 2).

### Activity in 3-D spheroid cell culture

**Figure 3:**
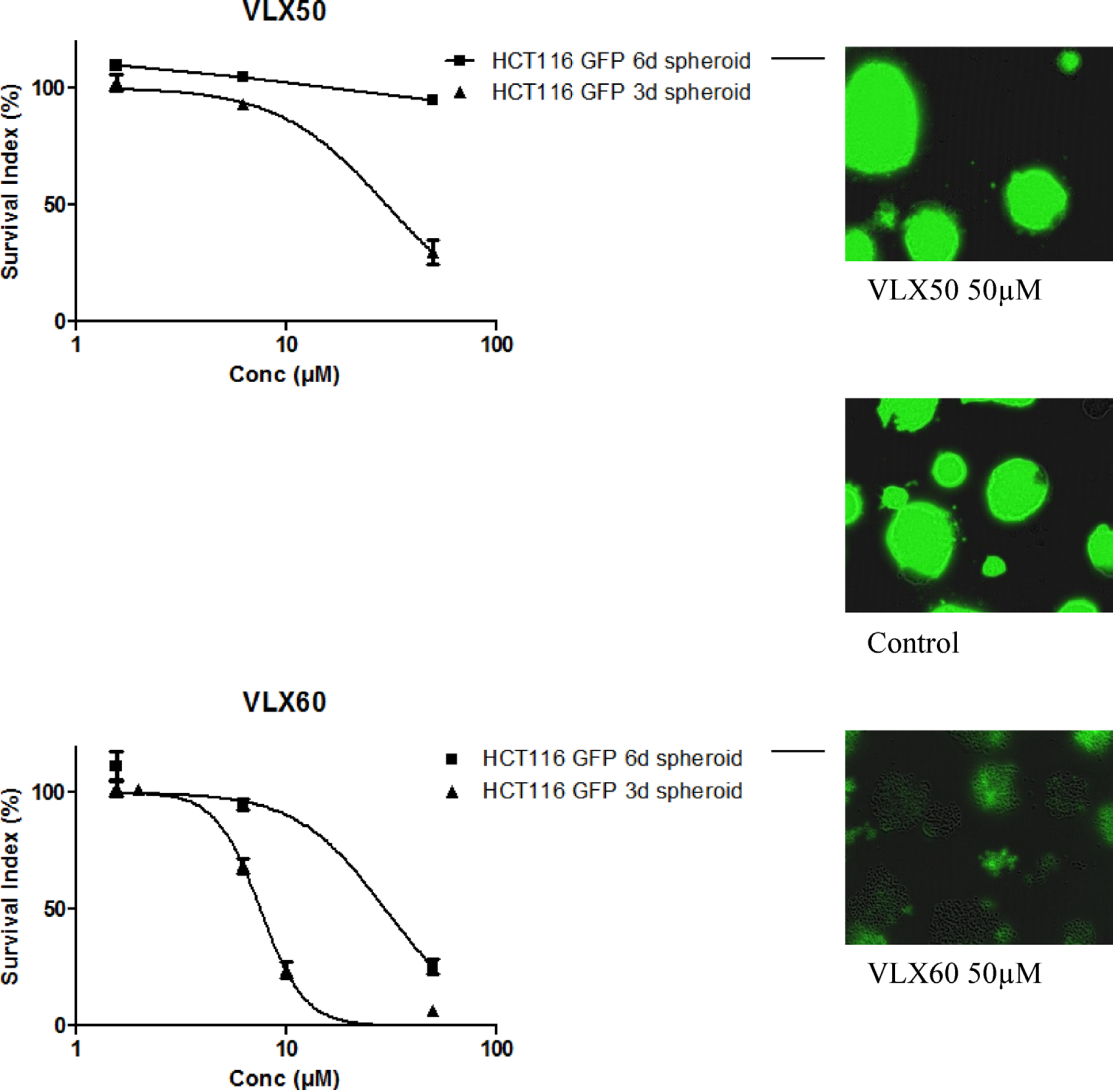
Cell survival in the FMCA assay, expressed as survival index of cell lines cultured as 3-D spheroids for 3 or 6 days and then exposed to VLX50 (upper panel) or VLX60 (lower panel) for 72 h Concentration-response curves (mean ± SEM) are based on three to six independent experiments with duplicate or triplicate wells for each concentration. Representative fluorescence images of 6 days spheroids exposed to VLX50, VLX60 and control are shown in the right panel. A concentration-response curve fitting was not possible for HCT116 GFP 6 days spheroids for VLX50 because of the configuration of the data.

VLX50 and VLX60 activity was then examined in a multicellular tumor spheroid (MCTS) model, considered to better reflect the solid tumor *in vivo* with respect to drug penetration, cell interactions, gene expression, hypoxia and nutrient gradients compared with monolayer cultured cells [33–35]. Both VLX50 and VLX60 were active in 3 day-old spheroids, although VLX50 was 4-fold less potent (Figure 3). Whereas 6 day-old spheroids were completely resistant to VLX50 at up to 50 μM, VLX60 exhibited an IC_50_ of less than 30 μM in this non-proliferative and very resistant tumor model. Fluorescence imaging revealed a clearly visible effect on 3- and 6 day-old spheroids after exposure to VLX60. This is demonstrated in Figure 3, where in addition to a lower fluorescence signal also a partial dissociation of 6 day-old spheroids is evident after exposure to 50 μM VLX60.

### Activity in tumor cells from patients

**Figure 4:**
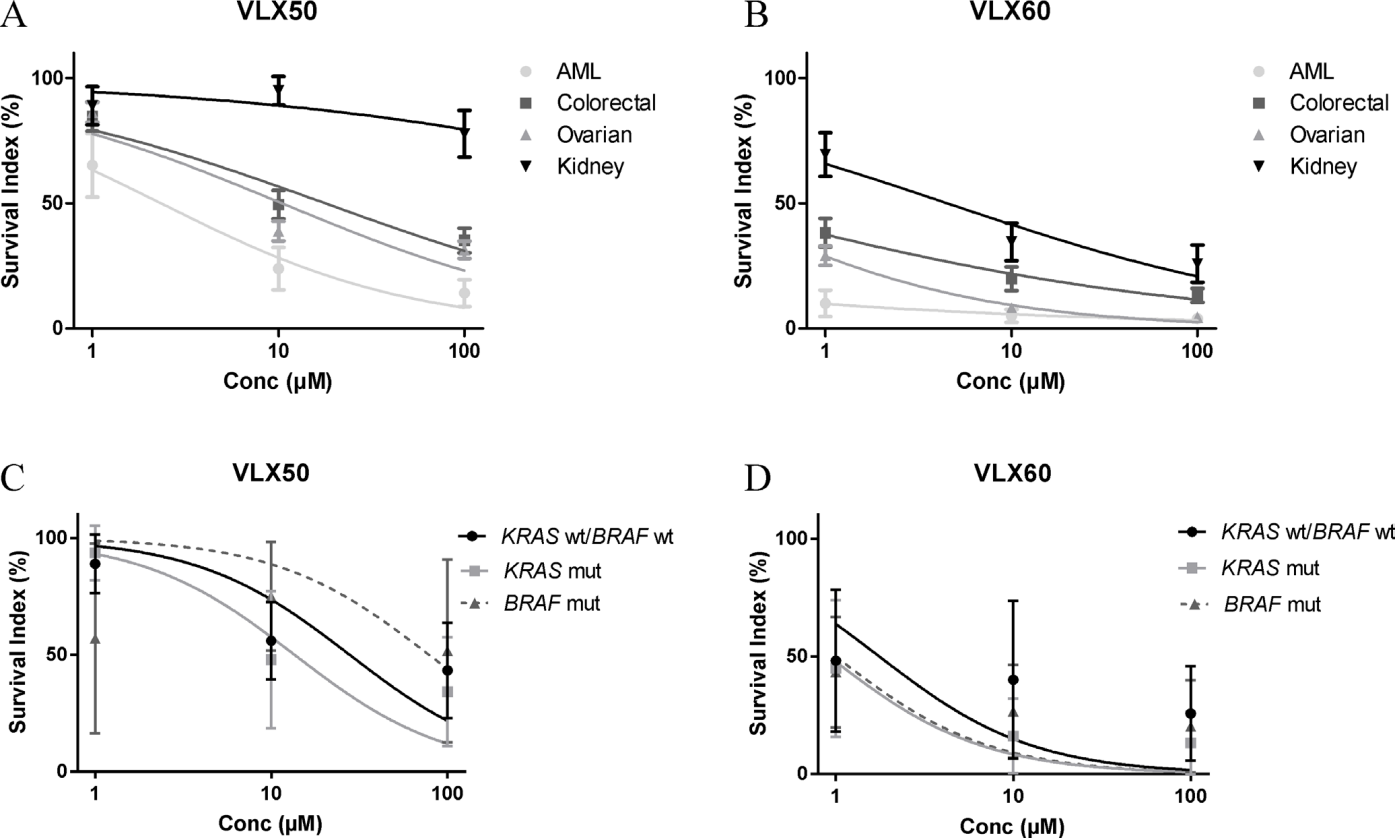
Cell survival in the FMCA assay, expressed as survival index, of patient tumor cells representing the indicated diagnoses when exposed to VLX50 (**A**) or VLX60 (**B**) for 72 h. Concentration-response curves (mean ± SEM) are based on 7 to 26 samples, with duplicate wells for each drug concentration. AML, acute myeloid leukemia. (**C**–**D**) Antitumor activity of VLX50 and VLX60 in patient tumor cells from patients with colorectal cancer divided by *KRAS* and *BRAF* mutation status. IC_50_-values are shown in Table 2. *KRAS* and *BRAF* mutation status was available in 16 colorectal cancer patient samples, all investigated for both drugs (*KRAS* wt/*BRAF* wt, *n = 6*; *KRAS* mut, *n = 7*; *BRAF* mut, *n = 3*). Concentration-response curves show means ± SEM.

Given the differential activity of both VLX50 and VLX60 against the HCT116 colon cancer cell lines vs. the other cell lines tested, the activity of these drugs was assessed in tumor cells from patients with colorectal cancer and compared with cells from patients with acute myeloid leukemia (AML), ovarian or kidney cancer (Figure 4A–4B). Similar patterns of VLX50 and VLX60 activity were demonstrated in the four malignancies with AML cells being the most sensitive, followed by ovarian, colon- and kidney cancer cells. Importantly, VLX60 showed a ≥ 40-fold higher cytotoxic activity than VLX50 (Figure 4A–4B, IC_50_-values not shown). Thus, although the activity pattern in the patient cells did not fully correspond to that in the cell lines, the relative effect of VLX60 compared to VLX50 was higher in tumor cells from patients than in the cell lines. As shown in Table 2 and Figure 4C–4D VLX60 exhibited a trend towards enhanced activity against both *KRAS* and *BRAF* mutated tumor cells from patients with colorectal cancer although the difference was not statistically significant (*KRAS* and *BRAF* mutation status was only available in 16 patient samples).

**Table 2:** Antitumor activity of VLX50 and VLX60 in patient tumor cells from patients with colorectal cancer

mCRC KRAS and BRAF mutation status	VLX50		VLX60	
	IC_50_ (μM)	95% CI	IC_50_ (μM)	95% CI
*KRAS* wt/*BRAF* wt	27.8	9.74, 85.8	1.75	0.39, 11.7
*KRAS* mut	13.5	5.87, 36.3	0.91	0.44, 1.79
*BRAF* mut	78.5	4.03, N/A	1.00	0.18, 4.65

*KRAS* and *BRAF* mutation status was available in 16 colorectal cancer patient samples (KRAS wt/BRAF wt, *n* = 6; KRAS mut, *n* = 7; BRAF mut, *n* = 3). Concentration-response curves are shown in Figure 4C–4D.

### Gene expression analysis

**Figure 5:**
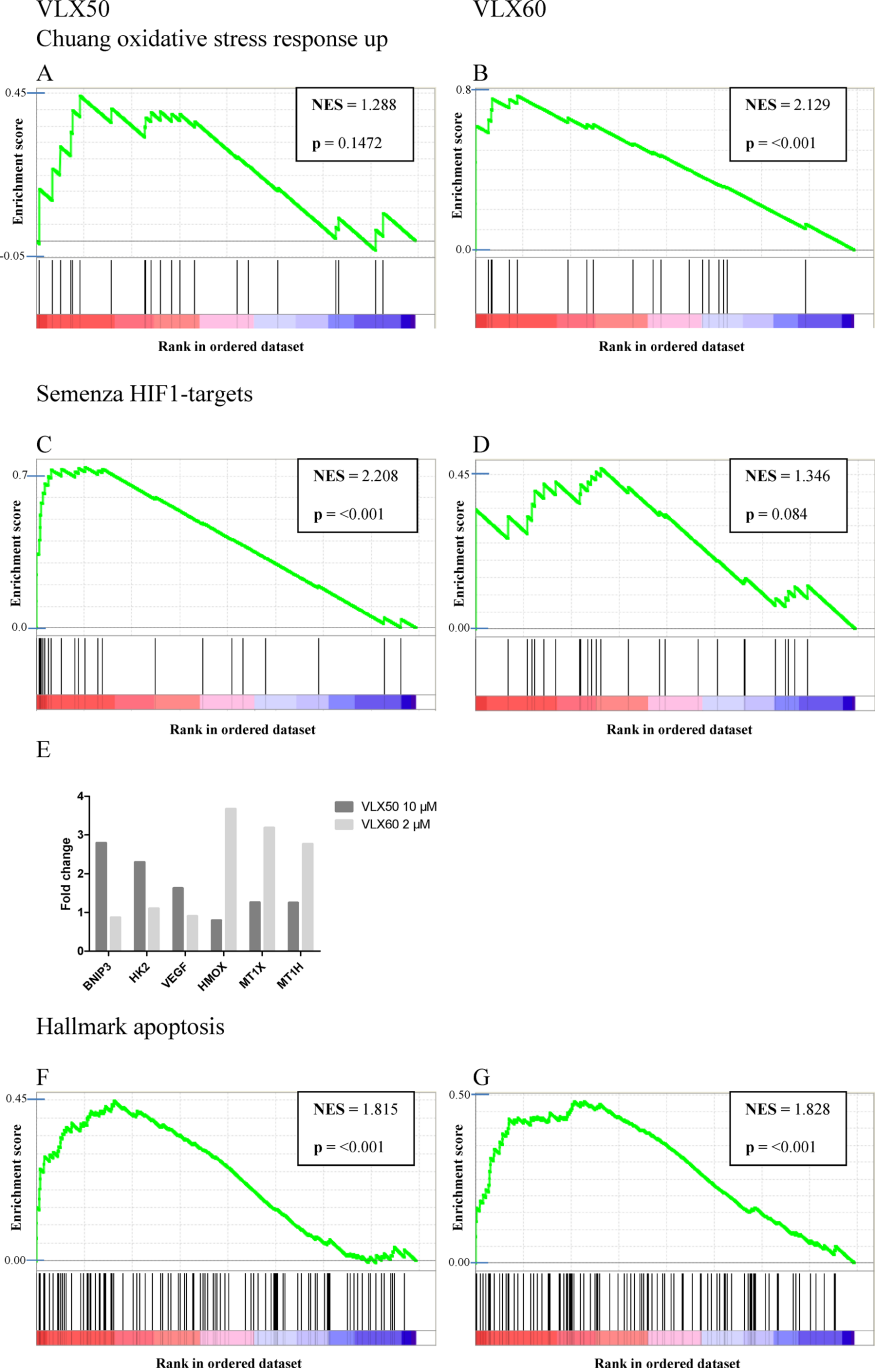
Gene set enrichment analysis (**A** and **B**) Up-regulation of genes associated with oxidative stress. (**C** and **D**) Up-regulation of genes associated with hypoxia inducible factor 1 (HIF1) signaling. (**E**) Fold change of key genes in the HIF1 signaling pathway (BNIP3, HK2 and VEGF) and genes associated with oxidative stress (HMOX, MT1X and MTX1H) as evaluated by the GSEA. (**F** and **G**) Up-regulation of genes associated with apoptosis. Concentrations used were VLX50 10 μM and VLX60 2 μM. The genes are rank ordered according to their relative expression. Enrichment profiles (green lines) are shown of genes (black vertical lines) in each gene set (red = positively correlated; blue = negatively correlated). NES, normalized enrichment score. Individual genes included in the a priori defined gene sets are presented as supplementary material in the excel-files named “Supplementary Table 1 (VLX50 10 μM) and Supplementary Table 2 (VLX60 2 μM)”.

The most substantial finding from the Gene Set Enrichment Analysis (GSEA) was that genes associated with oxidative stress were substantially enriched in cells exposed to VLX60 compared with VLX50 (Figure 5A–5B). Among 4 431 a priori defined gene sets, the gene set “Chuang oxidative stress response up” exhibited the highest enrichment score for VLX60 (Figure 5A). Furthermore, in accordance with earlier published results [5], the GSEA showed that genes associated with HIF1 signaling were induced by exposure to VLX50, as opposed to VLX60 (Figure 5C–5D). This hypoxia response associated with VLX50 treatment has been shown to be consistent with iron chelation as mechanism of action [5]. Hallmark genes associated with apoptosis were enriched in cells exposed to either drug (Figure 5F–5G). Individual key genes in the HIF1 signaling pathway and genes associated with oxidative stress are shown in Figure 5E.

### Oxidative stress and effects on the ubiquitin-proteasome system

**Figure 6:**
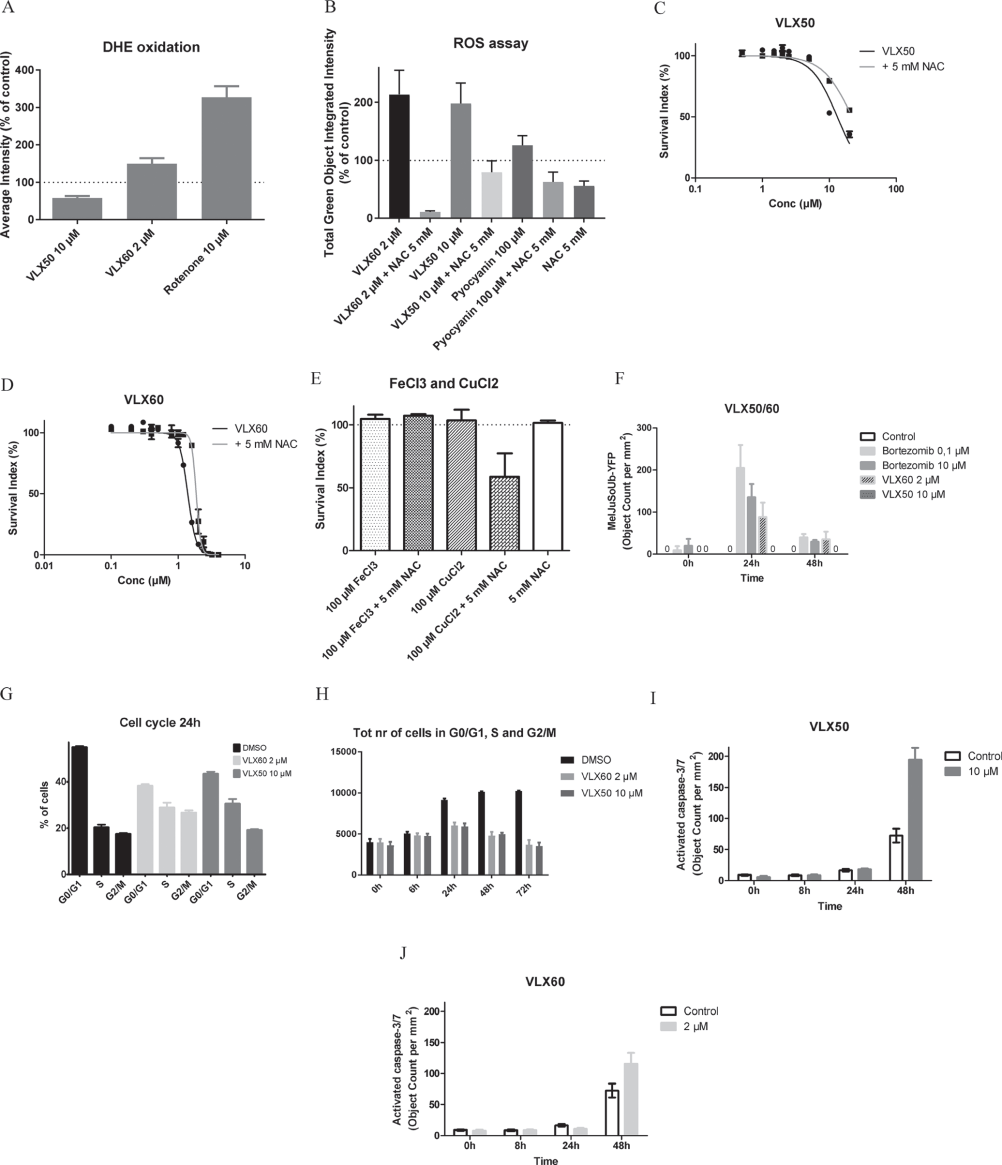
Oxidative stress, effects on the UPS, cell cycle and apoptosis (**A**) Induction of oxidative stress as judged by the assessment of the superoxide indicator dihydroethidium (DHE) oxidation in HCT116 cells after 24 h. Results are expressed as percentage of control based on six independent experiments, with single or duplicate wells for each drug concentration. Rotenone, a well-known inducer of oxidative stress, was included for comparison. (**B**) Induction of early reactive oxygen species (ROS) formation as judged by the assessment of the Oxidative Stress Detection Reagent in HCT116 cells after 2 h. The non-fluorescent probe has a low sensitivity for superoxide and reacts directly with a wide range of reactive species, *e.g*. hydrogen peroxide, peroxynitrite, hydroxyl radicals, nitric oxide and peroxy radical, yielding a green fluorescent product. Results are expressed as percentage of control based on three independent experiments, with quadriplicate wells for each drug concentration. The oxidative stress inducer pyocyanin was included for comparison. The ROS inhibitor NAC reverses induction of ROS formation in cells treated with VLX50 or VLX60. (**C**–**D**) The ROS inhibitor NAC reverses the effect of both VLX50 and VLX60 on HCT116 cells. Concentration-response curves (mean ± SEM) are based on four independent experiments with quadruplicate wells for each concentration. (**E**) The ROS inhibitor NAC interacts with CuCl_2_ to induce cell death. (**F**) Accumulation of ubiquitin-proteasome system (UPS) substrate Ub^G76V^-YFP induced by VLX60 as evaluated by the increase in fluorescent activity over time in MelJuSoUb-YFP melanoma cells transfected with a plasmid encoding the reporter substrate Ub^G76V^-YFP. Results are based on two independent experiments, with triplicate wells for each drug concentration. VLX50 showed no accumulation of UPS substrate Ub^G76V^-YFP. Bortezomib, a well-known proteasome inhibitor, was included for comparison. (**G**) Effect of VLX50 and VLX60 on cell cycle in HCT116 cells. (**H**) VLX60 inhibits cell proliferation at a lower concentration than VLX50. Results in G-H are based on three to four independent experiments, with single or duplicate wells for each drug concentration. (**I**–**J**) Induction of apoptosis as judged by the IncuCyte analysis of activated caspase-3/7 over time in HCT116 cells. Results are based on two independent experiments, with triplicate wells for each drug concentration.

Exposure to VLX50 for 24 h was not associated with ROS formation, as judged by the assessment of the superoxide indicator dihydroethidium (DHE) (Figure 6A). In contrast, an increase in ROS formation was noted 24 h after exposure to VLX60, although less than from the same concentration of rotenone, a well-known inducer of oxidative stress [36, 37]. ROS induction was also analyzed after 2 and 6 h drug exposure. In these experiments an increase in ROS by time was demonstrated with 2 μM and 10 μM VLX60, whereas there was no ROS induction with the same concentrations of VLX50 (not shown). Both VLX50 and VLX60 induced early ROS formation other than superoxide as judged by the assessment of the Oxidative Stress Detection Reagent in HCT116 cells after 2 h (Figure 6B). The oxidative stress inducer pyocyanin was included as positive control and induced ROS formation to a lesser degree than the experimental drugs, probably explained by a relatively low concentration of pyocyanin in a higher concentration of dimethyl-sulfoxide (DMSO) (known to inhibit ROS formation). The ROS inhibitor N-acetylcysteine (NAC) reversed induction of ROS formation in cells treated with VLX50, VLX60 or positive control (Figure 6B). Pre-incubation with the ROS inhibitor NAC resulted in a right-shift of the concentration-response curve for both VLX50 and VLX60 (Figure 6C–6D) whereas NAC interacted with CuCl_2_ to induce cell death (Figure 6E).

Whereas there was no sign of accumulation of the UPS substrate Ub^G76V^-YFP after exposure to VLX50 (not shown), exposure to VLX60 produced an increase in fluorescence after 24 h, similar to bortezomib, a known proteasome inhibitor (Figure 6F).

### Cell cycle, cell proliferation and apoptosis

Cell cycle analysis after 24 h exposure revealed no substantial difference between the two compounds; both accumulated cells in S and G2/M phases (Figure 6G). After 6 h drug exposure, VLX50 compared with VLX60 accumulated more cells in G1 (Online Resource, Supplementary Figure 4). Figure 6H confirms the equipotency of the VLX50 and VLX60 concentrations chosen for the various experiments as they inhibited cell proliferation up to 24 h to the same extent. After 24 h the number of cells available for analysis decreases, as a sign of cell death.

Both VLX50 and VLX60 induced apoptosis after 48 h at concentrations close to IC_50_-values (10 μM and 2 μM respectively), as judged by analysis of activated caspase-3/7 (Figure 6I–6J). VLX60 at higher concentrations (10 and 50 μM) elicited an almost immediate activation of caspase-3/7 in contrast to VLX50 (not shown). However, exposure to VLX60 at these higher concentrations was associated with a complete cell death already at 24h (not shown).

### Antitumor activity of VLX60 in xenograft model

**Figure 7:**
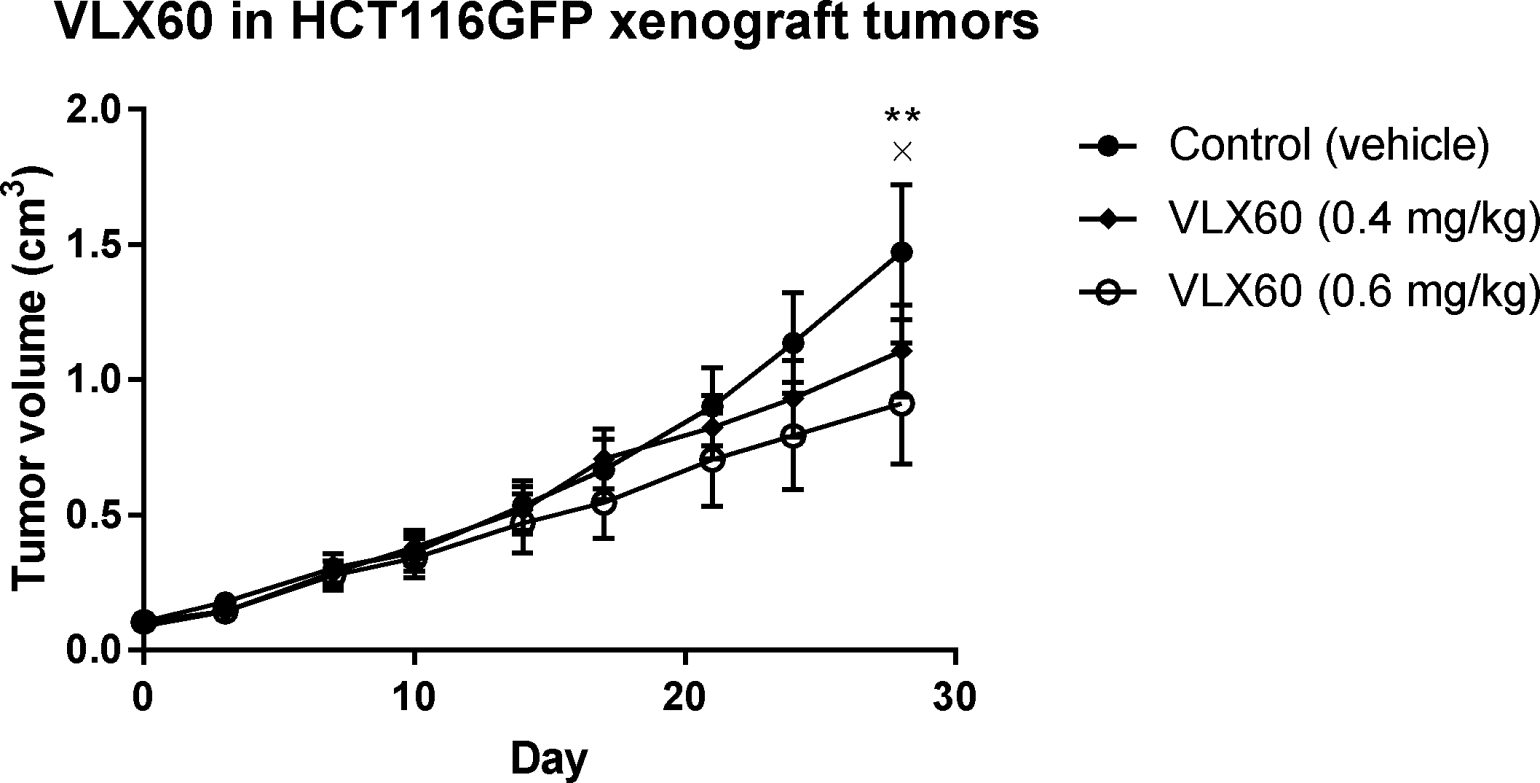
Effect of VLX60 *in vivo* Data presented show tumor volume over time in mice bearing xenografts of human colon cancer cell line HCT116 GFP following intraperitoneal administration of 0.4 or 0.6 mg/kg/day of VLX60. A 100 μl cell suspension containing 5 × 10^6^ HCT116 GFP cells were injected subcutaneously at the right rear flank of the animals on day -10. At inclusion into the study (day 0) the majority of the tumors had reached 0.1 cm^3^ or slightly above and the animals were divided into one of the three study groups (*n* = 10 per group). VLX60 was administered daily (0.08 or 0.12 mg/mL in NaCl with 4% DMSO) from day 0 to day 28. Vehicle control (5 mL/kg of 1% CMC in PBS with 8% DMSO) was administered orally (by gavage, bid) days 0-2 (only one administration was given on day 2). **= *P* = 0.0035 for VLX60 0.6 mg/kg *vs* control at day 28. × = *P* = 0.0968 for VLX60 0.4 mg/kg *vs* control at day 28. CMC = carboxymethylcellulose sodium salt. PBS = phosphate buffered saline. DMSO = dimethyl sulfoxide. Bid = twice a day.

The antitumor activity of VLX60 was explored in HCT116 GFP xenograft tumors in mice. Figure 7 shows that 0.6 mg/kg/day VLX60 inhibited tumor growth (*p* < 0.05) after 28 days, compared to control. In contrast, 0.4 mg/kg/day VLX60 did not significantly inhibit tumor growth (*p* > 0.05) after 28 days (Figure 7). VLX60 was well tolerated at both doses as assessed from animal pattern of gait and body weight. However, 6 animals (*n* = 1 in control group; *n* = 3 in 0.4 mg/kg/day VLX60 group; *n* = 2 in 0.6 mg/kg/day VLX60 group) were euthanized pre-term due to a wound or penetrating cavity on the tumor.

## DISCUSSION

The thiosemicarbazone family of drugs has been investigated for several decades after the antitumor activity of glyoxal-bis(thiosemicarbazones) was first established for Sarcoma 180 in Swiss mice in the 1950s [38, 39]. However, side effects, e.g. Triapine induced methemoglobinemia, have limited their potential clinical efficacy in solid tumors [15] and a rationally designed change of a known thiosemicarbazone into a more potent drug with a change in mechanism of action that utilizes tumor specific vulnerabilities, e.g. ROS and driver mutations, would be desirable.

Copper complexes of thiosemicarbazones are known for their redox-active properties and the induction of cell death by these compounds has been associated with a broad spectrum of mechanisms [8, 17–22]. The ability of copper chelates of thiosemicarbazones to induce cell death associated with generation of reactive oxygen species (ROS) and depletion of cellular glutathione was described early [17, 19]. However, most investigations of copper complexes have focused on the interaction with DNA [22]. Little is still known about the mechanisms of action and effects on intracellular signal transduction of copper complexes [22], although organelles such as the proteasome are emerging as new putative targets [22, 29–32]. Furthermore, copper complexes have been suggested to be able to overcome platinum resistance [17, 18, 22, 24, 26]. Although these interesting features of copper complexes of thiosemicarbazones have not yet materialized in the clinic and no copper complex of a thiosemicarbazone has thus far been approved for use against cancer, the interest in the development of copper complexes as anticancer agents has rapidly grown in the last decade [22].

The results presented here show that the copper complex VLX60 has properties worthy of further evaluation and is mechanistically different from the original compound VLX50. Firstly, VLX60 showed ≥ 3-fold higher cytotoxic activity than VLX50 in 2-D cultures from cell lines (Figure 2 and Table 1). This difference in activity was substantially more pronounced in tumor cells from patients (Figure 4). Also, the retained effect in the resistant spheroid model (Figure 3), makes VLX60 a promising drug candidate for further evaluation in solid tumors since three-dimensional cell cultures are thought to better reflect the situation *in vivo* in cancer patients with respect to drug interaction, cell-cell interactions, hypoxia and nutrient gradients in the tumor [33–35].

Next, we included colon cancer models able to associate the activity to the *KRAS-* and *BRAF* mutation status, established to have predictive and/or prognostic importance in this tumor type [27, 28]. The activity of VLX60 was significantly higher in RKO cells with mutant *BRAF* (Figure 2H and Table 1). The impact of a drug that could selectively target *BRAF*-mutated colorectal cancer would be of great clinical benefit, since *BRAF*-mutation is associated with a poor prognosis and drug resistance [28]. Interestingly, VLX60 exhibited a trend towards specific activity against both *KRAS-* and *BRAF* mutated tumor cells from patients with colorectal cancer (Figure 4C–4D and Table 2). Thus, further evaluation of VLX60 in *BRAF* mutated colorectal cancer is warranted.

KRAS and BRAF are both members of the MAPK-pathway, and it is known that mutational activation of this pathway can generate excess ROS [32, 40]. Since cancer cells in advanced tumors frequently exhibit high oxidative stress, an excessive ROS production through pharmacological insults has been proposed as an effective strategy to selectively eliminate these cancer cells [32]. As discussed below, induction of ROS formation is proposed to be important in the mechanism of action of VLX60. Thus, the selective effect against *BRAF* mutated RKO cells seen with VLX60 (Figure 2H and Table 1) with a trend towards such effect in patient cells with mutated *KRAS* or *BRAF* (Figure 4D and Table 2) are suggested to at least partly be due excessive ROS production in cells with altered redox regulatory mechanisms secondary to activation of the MAPK-pathway and the fact that *BRAF*-mutation status in RKO cells did not affect the response to VLX50 (Figure 2G and Table 1) could be due to a lower induction of ROS formation by VLX50 compared to VLX60 (see below).

Interestingly, gene expression analysis showed that genes that are upregulated by KRAS activation were upregulated after exposure to VLX50 in contrast to VLX60 (Online Resource, Supplementary Figure 5). Since superoxide can activate the kinases MEK and ERK downstream of KRAS and BRAF [41] and superoxide formation was induced by VLX60 in contrast to VLX50 (Figure 6A), a possible explanation is that ROS induced feed-back regulation in the MAPK-pathway is responsible for these differences in gene expression patterns. However, investigation of the underlying relationships between ROS signaling and the impact of KRAS-/BRAF signaling is beyond the scope of this study.

Of note, 72 h is a well-established incubation time (confluence of HCT116 cells in non-treated control wells stabilizes after ~72h) in our lab for assessment of drug induced cytotoxic effects and, therefore, the endpoints for mechanistic studies should be evaluated at earlier time points when the cells are still viable. Thus, early assessment of gene expression demonstrated that among 4 431 a priori defined gene sets, the most substantial finding was that genes associated with oxidative stress were considerably enriched in cells exposed to VLX60 (the gene set “Chuang oxidative stress response up” exhibited the highest enrichment score), in contrast to VLX50 (Figure 5A–5B). VLX60 induced ROS formation was confirmed by assessment of the superoxide indicator DHE (Figure 6A) and its importance by the reduction in cytotoxic activity after pre-incubation with the ROS inhibitor NAC (Figure 6D).

NAC is a precursor to glutathione with antioxidant properties but has also been described to act as a reducing agent that could activate copper complexes extracellularly and induce ROS formation when co-administered [24]. However, NAC pre-loading has instead been associated with protective effects against ROS generating copper complexes [24]. In our study, pre-incubation with NAC reduced the cytotoxic activity of VLX60 and therefore the interpretation is that a theoretically expected enhanced cytotoxic activity induced by a reduction of the copper complex extracellularly is outweighed by the antioxidant properties of NAC intracellularly. Importantly, in contrast to the combination NAC + VLX60, the combination NAC + 100 μM CuCl_2_ was toxic (Figure 6E) whereas either NAC or CuCl_2_ alone were non-toxic (Figure 6E). This is in accordance with previous published results which showed that the combination NAC + CuCl_2_ can induce formation of ROS extracellularly and subsequently induce oxidative stress in cells through transportation of ROS into the cell [42]. Furthermore, because 100 μM CuCl_2_ was non-toxic to cells (Figure 6E), anticancer activity by the copper complex VLX60 most probably is associated with biological activities of the copper complex, not simply from free copper shuttled into cells by VLX60.

A reduction of the cytotoxic activity after pre-incubation with NAC together with an induction of ROS other than superoxide were seen with VLX50 (Figure 6B–6C) and might be explained by the ability of VLX50 to scavenge Cu in the cell culture system and induce ROS production through formation of VLX60. Also, VLX50 could theoretically utilize accumulated copper in cancer cells as a means of inducing ROS at levels non-detectable by microarray analysis and assessment of DHE but at levels able to interfere with intracellular signaling. However, since the gene expression results (Figure 5A–5B) and the DHE assay (Figure 6A) demonstrate a clear involvement of ROS in the mechanism of action of VLX60 only, the reduced cytotoxic activity observed for both drugs after pre-incubation with NAC might also have other, yet unknown, explanations.

Overall it is not surprising that VLX60 is characterized by ROS production since this has previously been described as one of the primary ways in which Cu-complexes exert their effects [8, 22, 43, 44]. Supplementary Figure 3 suggests that VLX50 binds Cu and once formed, the complex enters the cells. Importantly, in concordance with results presented for other thiosemicarbazones VLX60 retained its activity in the presence of iron, whereas addition of iron impaired the cytotoxic activity of VLX50 [4, 5, 8] (Online Resource, Supplementary Figure 3). The addition of Cu enhanced the cytotoxic activity of both VLX50 and VLX60 (Online Resource, Supplementary Figure 3). CuCl_2_ or FeCl_3_ were nontoxic to HCT116 cells and the addition of CuCl_2_ and FeCl_3_ resulted in no change in the IC_50_-value of the standard drug 5-FU (Online Resource, Supplementary Figure 3). Altogether, these data suggests that VLX50 can react with Fe in contrast to VLX60 and that VLX60 undergoes reductive dissociation in cells, releasing Cu, and then can pick up supplemental Cu to enhance the overall accumulation of Cu in cells. Also, the decrease in gene expression of genes associated with cation channel activity after exposure to VLX60 as opposed to VLX50 (Online Resource, Supplementary Figure 5) suggests a negative regulation secondary to an effective transport of copper intracellularly after exposure to VLX60.

Thiosemicarbazones have recently been described as possible ionophores for metal ions [45]. However, VLX50 can be considered an ionophore only if VLX60 is coupled into redox chemistry that reduces Cu(II) to Cu(I), reducing its affinity for VLX50 and subsequently making it available to react within the cell. Also, since thiosemicarbazones have been shown to interact not only with copper and iron but also with other metal ions, such as zinc [8], it is possible that VLX60 might exchange other metal ions with copper intra- or extracellularly. The possible interaction of thiosemicarbazones with intracellular ions and organelles is indeed an interesting subject for further studies. Recently, antitumor activity of the copper complex of the thiosemicarbazone Dp44mT was coupled to oxidative stress formation through accumulation of the redox active copper complex in acidic lysosomes [46] and cytotoxicity was greater against resistant cells than their nonresistant counterparts [3]. Also, since cancer cells have been shown to exhibit higher copper levels than normal cells [43, 47], an excessive copper load through pharmacological insults has been proposed as an effective strategy to selectively eliminate these cancer cells. The results presented here indicate that iron depletion is of less importance for the mechanism of action of VLX60 compared to VLX50 but that the interaction with copper is of potential interest for the cytotoxic activity of both VLX50 and VLX60 (Online Resource, Supplementary Figure 3). Although data suggests that VLX50 can both pick up Fe and compete for Cu to form VLX60 whereas VLX60 can probably not react with Fe (Online Resource, Supplementary Figure 3), a very high and non-physiological extracellular concentration of copper would probably need to be available for VLX50 to accumulate Cu intracellularly in a similar way as VLX60.

Since proteasome inhibition has emerged as a putative target for copper complexes we also evaluated the effect of VLX60 on the ubiquitin-proteasome system (UPS) [22, 29–32]. In MelJuSoUb-YFP experiments, VLX60 exposure showed an increase in fluorescence after 24 h similar to bortezomib, an established proteasome inhibitor (Figure 6F). However, the MelJuSoUb-YFP assay indicates compromise of the UPS rather than specific proteasome inhibition. Interestingly, proteasome inhibition has recently been suggested to represent a valuable target strategy in *BRAF*-mutant colorectal cancer [48]. Thus, involvement of BRAF in the mechanism of action of drugs that compromise the UPS needs to be further elucidated.

Important general features of cytotoxic drugs such as effects on cell cycle, cell proliferation and apoptosis were assessed. No substantial difference between the two compounds was observed in the cell cycle analysis at 24 h (Figure 6G). Whereas VLX60 was more potent than VLX50, both compounds inhibited cell proliferation in HCT116 cells up to 24 h, after which total cell number decreased as a sign of cell death (Figure 6H). Both VLX50 and VLX60 were associated with apoptotic cell death as judged by the gene expression analysis and increase in fluorescence reporting caspase-3/7 (Figure 6I–6J). However, the apoptosis process was faster for VLX60, as indicated by the low survival and earlier increase in fluorescence reporting caspase-3/7 after exposure to higher concentrations of VLX50 and VLX60 (not shown).

In previous studies, thiosemicarbazones were proposed to induce apoptosis through restoration of mutated p53 [49, 50]. However, the almost identical concentration - response curves regardless of *p53*-status in HCT116 cells in this study argues against a role of p53 for the effect of VLX60.

Finally, the antitumor activity seen in the murine xenograft model (Figure 7) indicates that VLX60 can exert activity against malignant cells while sparing normal cells *in vivo*. This presence of a therapeutic window (a drug concentration range which is effective against tumors while sparing normal tissue) is essential in the treatment of cancer patients in the clinic and is supportive for the development of VLX60 into an anticancer drug. VLX60 treatment at the highest concentration was well tolerated and opens for testing of higher concentrations and different administration schemes to optimize tumor responses.

In conclusion, we demonstrate that VLX60 is considerably more potent than VLX50 and that the mechanism of action is different although both drugs are effective inhibitors of cell proliferation and induce apoptotic cell death. Of note, VLX60 is active also in non-proliferative models that better reflect the situation in cancer patients and cells derived from tumors in patients and, importantly, VLX60 exerts antitumor activity *in vivo*. Thus, VLX60 shows interesting properties of potential interest for development into a new anticancer drug, notably against *BRAF* mutated colorectal cancer.

## MATERIALS AND METHODS

### Ethics statement

This investigation was conducted in accordance with the ethical standards of the Declaration of Helsinki and national and international guidelines and was approved by ethical review boards as detailed below.

### Cell lines

The colon cancer cell line HC116 GFP was from Anticancer Inc. (San Diego, CA, USA). The kidney cancer cell lines ACHN, Caki-2, 786-O, the monocytic cell line U937-GTB and the normal colon cell line CCD 841 CoN were from American Type Culture Collection (ATCC; Manassas, VA, USA). The ovarian cancer cell line A2780 was obtained from European Collection of Cell Cultures (ECACC; Salisbury, UK). Three pairs of colon cancer cell lines were used to study the relationship between drug effect and *KRAS*/*BRAF* mutation status: HCT116, HCT116 *KRAS* (+/−) (KO of *KRAS* mutant allele in heterozygous parental cell), RKO, RKO *BRAF* (+/−/−) (KO of both mutant alleles in parental cell), DLD, DLD *KRAS* (+/−) (KO of *KRAS* mutant allele in heterozygous parental cell) were from Horizon Discovery Ltd., Cambridge, UK. All purchased cell lines were authenticated by short-term repeat analysis performed by the cell banks.

The colon cancer cell line HCT116 *p53*−/− was kindly provided by Prof. Bert Vogelstein (Johns Hopkins University, Baltimore, MD, USA). The melanoma cell line MelJuSoUb-YFP was kindly provided by Prof. Nico Dantuma (Karolinska Institute, Stockholm, Sweden).

The cell lines were cultured at 37°C in a humidified incubator containing 5% CO_2_ in McCoy’s 5A medium (Caki-2, HCT116, HC116 GFP, HCT116 *p53*−/−, HCT116 *KRAS* (+/−), RKO, RKO *BRAF* (+/−/−), DLD, DLD-*KRAS*(+/−)), RPMI1640 medium (A2780, U937-GTB, ACHN, 786-O), MEME medium (MelJuSoUb-YFP) or EMEM medium (CCD 841 CoN) supplemented with fetal calf serum and with change of medium as recommended. All media were from Sigma-Aldrich (St. Louis, MO, USA) except the EMEM medium which was from ATCC. Morphology and growth of cells were monitored on a weekly basis and all cell lines were passaged for less than 6 months.

### Patient tumor cells

Tumor samples were obtained from patients diagnosed with acute myeloblastic leukemia (AML), ovarian-, colorectal- or kidney cancer by bone marrow/blood sampling, surgery or diagnostic biopsy. The patient sampling was approved by the regional ethical committee, Uppsala University (file Dnr 2007/237). Tumor cells from bone marrow/blood (AML) were collected by centrifugation and isolated by Ficoll-Paque (GE Healthcare, Waukesha, WI, USA) and/or Percoll (GE Healthcare) density gradient centrifugation [51]. Samples from solid tumors were finely minced and digested with collagenase and tumor cells were isolated by Percoll (GE Healthcare) density gradient centrifugation [51]. *KRAS-* and *BRAF* mutation status of colorectal cancer patient samples was retrieved from pathology reports in the patient files.

### Drugs

VLX50 is a thiosemicarbazone experimental drug with iron depletion as main mechanism of action [5]. VLX60 is a copper chelate of VLX50. VLX50 was from Maybridge (Cambridge, UK) and VLX60 was synthesized by author GW. Since VLX60 has VLX50 as precursor a synthesis procedure to give access to larger amounts of VLX50 was developed. For synthesis and chemical characterization of compounds, see supplementary section 1 and Supplementary Figure 1 (Online Resource). Rotenone, CuCl_2_ and FeCl_3_ were from Sigma-Aldrich and bortezomib from LC Laboratories (Woburn, MA, USA). The compounds were dissolved in dimethyl-sulfoxide (DMSO) and diluted in either phosphate buffered saline (PBS) or distilled water, depending on solubility. N-acetylcysteine (NAC) was dissolved in distilled water. Final concentration of DMSO in experimental wells was always < 1%. Drug concentrations of VLX50 and VLX60 for the experimental comparisons were those inducing similar degrees of toxicity in HCT116 cells (10 μM VLX50 vs 2 μM VLX60).

### Measurement of cytotoxicity in monolayer cultured cell lines and patient tumor cells

The initial cell line experiments were done using 96-well microplates (Nunc, Roskilde, Denmark) prepared as previously described [52] with 20 μl drug solution at 10× the desired final concentration, using a multipipette or the pipetting robot BioMek 2000 (Beckman Coulter, Brea, CA, USA). The plates were kept at −70°C and thawed immediately prior to further use. On the first day of the experiment, 180 μl cell suspension with 5 000–20 000 cells (depending on the individual cell line doubling time) were seeded into each well in the drug-prepared 96-well plates, manually or by using the pipetting robot Precision 2000 (Bio-Tek Instruments, Winooski, VT, USA), reaching a final volume in each well of 200 μl.

For subsequent experiments in the colon cancer cell lines with different *KRAS*, *BRAF* or *p53* status (see above), cell suspension (2 500 cells/well) was added to drug-prepared 384-well plates as described below for patient samples or cells (2 500/well in 50 μl medium) were seeded into 384-well plates and allowed to pre-incubate overnight after which drug was added using the liquid handling system ECHO^®^ 550 (Labcyte Inc., Sunnyvale, CA, USA). This allows for fast transfer of volumes ≥2.5 nL from source plates into destination wells. In ECHO^®^ experiments, source plates were prepared with appropriate concentrations of drugs in DMSO and stored in the oxygen and moisture free MiniPod™ system (Roylan Developments Ltd, Surrey, UK) until further use. The cells were always incubated with drug for 72 h before assessment of cell viability (see below).

For tumor cells from patient samples, 45 μl cell suspension (5 000 cells/well for solid tumors, 40 000 cells/well for AML) were seeded into each well in the drug-prepared 384-well plates (5 μl/well of drug solution at 10x final concentration). All culture plates were incubated for 72 h at 37°C in 5% CO_2_ before assessment of cell viability.

Following the 72 h incubation, cell viability was assessed using the fluorometric microculture cytotoxicity assay (FMCA). The FMCA is based on the conversion of fluorescein diacetate (FDA, Sigma-Aldrich, cat. no. F7378) to fluorescein by esterases in cells with intact plasma membranes [51–53]. Briefly, cells were washed in PBS and FDA buffer [52] and FDA solution (0.5 mg ml^−1^ in DMSO) added (50 μl FDA buffer/well, 1 μl FDA solution/well). After 50–70 min incubation at 37°C, plates were read in the scanning fluorometer FLUOstar Optima (BMG Labtech GmbH, Offenburg, Germany). The assay was executed in a semi-automated robot system as previously described [52]. Cell viability as reported from the FMCA is expressed as survival index (SI) and is defined as the fluorescence in experimental wells (with blank wells subtracted) in per cent of that in control wells, with blank wells subtracted.

### Assessment of drug effects in tumor cell spheroids

Spheroids were prepared as described [54]. Briefly, 200 μl cell suspension with 5 000 HCT116 GFP cells were seeded into each well of a 96-well NanoCulture^®^ plate (SCIVAX USA, Inc., Woburn, MA, USA). The plates were incubated at 37°C in 5% CO_2_. Drug exposure started when the spheroids had formed for 3 or 6 days. At start of drug exposure, 100μl of medium/well was removed with a Multiwasher (DYNEX Technologies, Inc., Chantilly, VA, USA), followed by addition of 80 μl fresh medium and 20 μl drug solution at 10× the final concentration.

The spheroids were incubated with drugs for 72 h and then dissociated by addition of 100 μl/well of Accumax (PAA, Pasching, Austria) and incubation at 37for 30 min followed by mixing with a multipipette [54]. After one wash in PBS, cell viability was assessed as described above. The plates were also monitored in the IncuCyte fluorescence (FLR) system (Essen BioScience, Ann Arbor, MI, USA) [54], which allows for visual and functional quality control of spheroid growth through automated data acquisition of phase contrast and fluorescent images within the cell culture incubator prior to survival assessment with the FMCA.

### VLX 50 and VLX60 mechanistic exploration using gene expression analysis

The gene expression analysis of VLX50 and VLX60 was performed according to the original protocol, as previously described [55]. Briefly, HCT116 cells (0.3 × 10^6^ cells/well) were seeded into each well in 6-well plates and allowed to attach for 24 h. VLX50, VLX60 or vehicle control (DMSO) were then added at a final concentration of 10 μM (VLX50) or 2 μM (VLX60). Cells were incubated with drug or vehicle control for 6 h and then washed with PBS. RNA was isolated using RNeasy Mini Kit (Qiagen, Hilden, Germany). RNA concentration and quality were measured using an ND 1000 spectrophotometer (NanoDrop Technologies, Wilmington, DE, USA) and Bioanalyzer system (Agilent Technologies Inc., Palo Alto, CA, USA), respectively. Starting from 2 μg of total RNA from each sample, gene expression analysis was performed using Human Genome U133 Plus 2.0 Arrays according to the GeneChip Expression Analysis Technical Manual (Rev. nr 5, Affymetrix Inc., Santa Clara, CA, USA). Raw data was MAS5 normalized.

Gene expression ratios for drug vs. vehicle exposed cells were calculated to generate a list of regulated genes, based on the RMA normalized data, and this list was filtrated using flags from the MAS5 normalization. Only probes with present call in both drug treated and vehicle control lists were used in the Gene Set Enrichment Analysis (GSEA), as described previously [56]. Average fold change was used as rank metric for drug vs. vehicle exposed cells to establish the rank lists. The rank lists were compared with a priori defined and curated gene sets with the purpose to find out whether these a priori defined gene sets were significantly enriched toward the upper or lower end of the ranked lists. The *p*-value refers to the nominal *p*-value after 1000 permutations. Individual genes included in the a priori defined gene sets are presented as supplementary material in the form of excel-files named “Supplementary Table 1 (VLX50 10 μM)” and “Supplementary Table 2 (VLX60 2 μM)”. Raw and normalized expression data have been deposited at Gene Expression Omnibus (https://www.ncbi.nlm.nih.gov/geo/) with accession number GSE86309.

### Assessment of oxidative stress

*I*. HCT116 cells (5 000 – 20 000/well) were seeded into 96-well plates and allowed to pre-incubate overnight. Drugs were added the next day and the commercially available Cellomics^®^ Oxidative Stress I Kit (Thermo Fisher Scientific, Gothenburg, Sweden) was used to study oxidative stress. The assay was analyzed in the fluorescence microscope Arrayscan II high content screening system (Cellomics Inc., Pittsburgh, PA, USA). Fluorescence provides a readout of the superoxide indicator dihydroethidium (DHE) oxidation of ROS generation. *II*. Induction of other ROS than superoxide was assessed with Oxidative Stress Detection Reagent (Green) and the positive control pyocyanin from the Cellular ROS/Superoxide Detection Assay Kit (Abcam, Cambridge, UK). HCT116 cells (2500/well) were seeded into 384-well plates and allowed to pre-incubate overnight. NAC (5 mM) was added 30 min before drug. Drugs and Oxidative Stress Detection Reagent (Green) were added using the liquid handling system ECHO^®^ 550. The plates were incubated for 2 h, centrifuged and washed twice in PBS and then analyzed in the IncuCyte ZOOM Live-Cell Analysis System (Essen BioScience, Ann Arbor, MI, USA). Fluorescence provides a readout of the probe which has a low sensitivity for superoxide and reacts directly with a wide range of reactive species, *e.g*. hydrogen peroxide, peroxynitrite, hydroxyl radicals, nitric oxide and peroxy radical, yielding a green fluorescent product. *III*. In the ROS inhibitor experiments measurement of cytotoxicity was performed in 384-well plates (2 500 cells/well) as described above (with FMCA) with 5 mM NAC added 30 min before drug.

### Cell cycle analysis

HCT116 cells (0.3 × 10^6^ cells/well) were seeded into each well in 6-well plates and allowed to attach for 24 h. VLX50, VLX60 or vehicle control (DMSO) were then added at a final concentration of 10 μM (VLX50) or 2 μM (VLX60). Cells were incubated with drug or vehicle control for 0–72h and the commercially available kit Two-step cell cycle analysis (Application note No. 0254. Rev. 1.1; ChemoMetec, Allerod, Denmark) was used to study the cell cycle. The assay was analyzed in the NucleoCounter^®^ NC-250™ (ChemoMetec).

### Assessment of the effects on the ubiquitin-proteasome system and apoptosis

The IncuCyte FLR system (Essen BioScience) was used to study effects on the ubiquitin-proteasome system (UPS) and apoptosis. MelJuSoUb-YFP (yellow fluorescent protein) is a melanoma cell line transfected with a plasmid encoding the reporter substrate Ub^G76V^-YFP. Proteasome inhibition, endoplasmic reticulum (ER) stress or other stress factors compromise the UPS and cause accumulation of the UPS substrate Ub^G76V^-YFP. This accumulation results in an increase in cellular fluorescence [57]. MelJuSoUb-YFP cells were seeded at a concentration of 6 000 cells/90 μl MEME-medium/well into 96-well plates and allowed to pre-incubate overnight after which 10 μl drug solution at 10x the final concentration were added to each well. The plates were monitored in the IncuCyte for 48 h and the IncuCyte FLR system software was used to calculate object count per mm^2^ based on fluorescence.

For assessment of apoptosis, HCT116 cells were seeded at a concentration of 10 000 cells/80 μl McCoy’s medium/well into 96-well plates and allowed to pre-incubate overnight followed by addition of 10 μl/well of Essen CellPlayer^TM^ Kinetic Caspase-3/7 Apoptosis reagent drug (Essen) and then immediately 10 μl/well of drug at 10× the final concentration, reaching a final volume of 100μl/well. The final concentration of the kinetic apoptosis reagent was 2.5 μM. The plates were monitored in the IncuCyte for 48 h and the IncuCyte FLR system software was used to calculate object count per mm^2^ based on fluorescence. The assay provides a kinetic readout of apoptotic signaling based on activation of Caspase-3/7.

### Assessment of drug effects in tumor xenografts

Mice experiments received ethics approval by regional animal experimental ethics committee in Stockholm (North), approval number N37/15 and N188/15. The age of the mice were 7 weeks at arrival and acclimatisation was a minimum of five days before commencement of the experiment. Five animals per cage were maintained in individually ventilated cages (type IVC2). Temperature was kept at 21–22°C, humidity was 50–60% and a 12 h light, 12 h dark cycle was applied. Autoclaved tap water was available *ad libitum* in water bottles and the animals received R70, irradiated, diet (Lantmännen, Sweden). Female NMRI nu/nu mice (Crl:NMRI-Foxn1nu) from Charles River, Germany, were injected subcutaneously at the right flank with 5 × 10^6^ HCT116 GFP cells 10 days before randomization into treatment groups. When the majority of the tumors had reached 0.1 cm^3^ (day 0) the animals were randomly assigned into treatment groups (*n* = 10 animals per group) and administered a daily intraperitoneal dose (0.4 mg/kg or 0.6 mg/kg; 0.08 or 0.12 mg/mL respectively in NaCl with 4% DMSO) of VLX60 for 28 days or control vehicle (administered by oral gavage with 5 mL/kg of 1% carboxymethylcellulose sodium salt (CMC) in PBS with 8% DMSO twice daily from day 0–2; only one administration day 2). The length and width of each tumor was measured with the use of a caliper twice weekly and tumor volume was calculated by the formula length (cm) × width (cm) × width (cm) × 0.44. Body weight was recorded at the start and at the end of the study and the animals were checked daily for change in activity and appearance as signs of a change in general health status.

### Data analysis and presentation

For *in vitro* studies IC_50_ values, *i.e*. the concentration resulting in a SI-value of 50%, were obtained using non-linear regression in GraphPad Prism 5 (GraphPad Software Inc., CA, USA). Results are presented as means ± SEM or means ± 95% Confidence Interval (CI) for the number of experiments indicated. Comparisons between mutated- and parental cell lines were done with unpaired Student’s *t*-test. GraphPad Prism 5 was used for result calculations, statistical inference and graphical presentation.

For *in vivo* studies the mean with SEM was calculated for tumour volumes recorded during the experiment. At start, all groups comprised 10 animals (*N* = 10), but along the study a few animals were euthanized due to reduced health status. Tumour data from animals terminated pre-term was carried forward in the statistical analysis (one animal in the 0.4 mg/kg/day group was euthanized at day 3 due to abnormal gait and was excluded from the analysis). Differences in tumour volume between the groups were calculated using repeated measures (from day 0 to 28) two-way ANOVA followed by Tukey’s multiple comparisons test using GraphPad Prism 7. Comparisons were made between Group 1 (Vehicle) *vs* Group 2 (Vehicle + VLX60 0.4 mg/kg/day) and Group 1 (Vehicle) *vs* Group 3 (VLX60 0.6 mg/kg/day). A *p*-value of < 0.05 was used to indicate statistical significance.

## SUPPLEMENTARY MATERIALS FIGURES AND TABLES







## Abbreviations

Abbreviations are explained when first mentioned in the text.
