# Supplementary material for: Mechanistic characterization of a copper containing thiosemicarbazone with potent antitumor activity
Source: Oncotarget. 2017 Mar 17;8(18):30217–34. doi: 10.18632/oncotarget.16324 (PMC5444738; doi:10.18632/oncotarget.16324)
Supplement: Supplementary file 1 [file oncotarget-08-30217-s001.pdf]

Mechanistic characterization of a copper containing thiosemicarbazone with potent antitumor activity

Supplementary Materials

SUPPLEMENTARY MATERIALS AND METHODS

1.1 Synthesis of VLX60

^{1}H NMR and 13C NMR were recorded at 25°C on a Varian MR-400.

{[(methylsulfanyl)methanethioyl]amino}amine[1]

Prepared according to a slightly modified procedure Crushed KOH (11.4 g, 0.2 mol) was dissolved in EtOH (abs, 70 ml) and kept in an ice bath to maintain a reaction temperature below 5°C. Hydrazine hydrate (10.0 g, 0.2 mol) was added slowly to the mixture. CS_2 was added drop wise to the cooled reaction and stirred for an additional hour after complete addition of CS_2. The solvent was decanted off and the white residue was dissolved in 40% aqueous ethanol (60 ml). The reaction mixture was once again cooled to 5°C and Methyl iodide (20.8 g, 0.2 mole) was added slowly to the reaction mixture. A precipitate was formed that was filtered off and washed three times with distilled water.

The product was dried and the yield of product was 8.5 g, 0.07 mol, 34% All spectroscopic data in agreement with published data.

{[(methylsulfanyl)methanethioyl]amino}[(pyridin-2-yl)methylidene]amine

Methyl carbodithionate (2.2 g, 18.3 mmol) was suspended in 2-propanol (10 ml). To the suspension was 2-pyridinealdehyde (2.1 g, 18.7 mmol) added dropwise. The turbidity of the mixture increases drastically upon addition of the aldehyde to the reaction. An additionally 10 ml of 2-propanol is added and the mixture is stirred for 1.5 hours. The reaction is filtered and washed with cold 2-propanol. Recrystallization from Acetonitrile, The product is dried. Yield crude product: 3.49 g, 16.5 mmol, 90.2%.

^{1}H NMR ($CDCl_3$ 400 MHz) In $CDCl_3$ the molecule exist in two conformations giving double sets of signals.

8.74 ppm, dt *J* = 1, 4 Hz, (1H) 8.62 ppm, dt *J* = 1, 8 Hz, (1H), 8.06 ppm, d *J* = 8Hz, (1H), 7.93 ppm, s, (1H), 7.87 ppm, dt, *J* = 1, 8 Hz (1H), 7.74 ppm, dt, *J* = 1, 8 Hz (1H), 7.47 ppm, d, *J* = 8 Hz, (1H), 7.37 ppm, ddd, *J* = 1, 4, 8 Hz, (1H), 7.30 ppm, ddd, *J* = 1, 4, 8 Hz, (1H), 7.28 ppm, s, (1H), 2.66 ppm, s, (3H)

3-(3-methoxypropyl)-1-[[(pyridin-2-yl)methylidene]amino]thiourea (CuCl2 VLX50)

Hydrazinecarbodithioic acid, 2-(2-pyridinylmethylene)-,methyl ester (1.0 g, 4.7 mmol) and 3-methoxypropylamine (0.6 g, 6.5 mmol) was suspended in Methanol (8.6 g) and was refluxed for 22 hours. During the reaction the reaction mixture turned yellow. The crude product was recrystallized from Acetonitrile. Yield 0.41, 1.5 mmol, 33.7%.

^{1}H NMR and 13C NMR spectra of the intermediate VLX50 were recorded:

^{1}H NMR (CDCl3 400 MHz) 9.59 ppm, s, (1H), 8.60 ppm, d, *J* = 4 Hz, (1H), 8.21 broad singlet (1H), 7.89 ppm, (1H), 7.86 ppm, d, *J* = 8 Hz, (1H), 7.69 ppm, ddd, *J* = 1, 8, 8 Hz, (1H), 7.26 ppm, ddd, *J* = 1, 8, 8 Hz, (1H), 3.82 ppm, t, *J* = 4 Hz, (2H), 3,57 ppm, t, *J* = 4 Hz (2H), 3.38 ppm, s, (3H), 1.94 ppm, tt, *J* = 4 Hz (2H).

13C NMR (CDCl3 100 MHz) 177.41, 152.76, 149.71, 141.84, 136.38, 124.13, 120.31, 72.20, 58.85, 43.96, 28.42.

Copper(II) chloride complex of 3-(3-methoxypropyl)-1-[[(pyridin-2-yl)methylidene]amino]thiourea (VLX60)

To Hydrazinecarbodithioic acid, 2-(2-pyridinylmethylene)-,3-methoxypropyl ester, VLX 50, (0.30 g, 1.18 mmol) in 15 ml EtOH was $CuCl_2$ (0.20 g, 1.17 mmol) in EtOH 6 ml added. Upon addition of the green copper solution the reaction mixture turns dark and after 2 minutes the precipitation starts and the reaction solution turns green. The reaction was stirred at room temperature for 4 hours filtered and washed with EtOH. Yield: 380 mg 1 mmol, 85%.

Suggested mechanism of VLX60 formation, first by addition of $CuCl_2$ to VLX50:

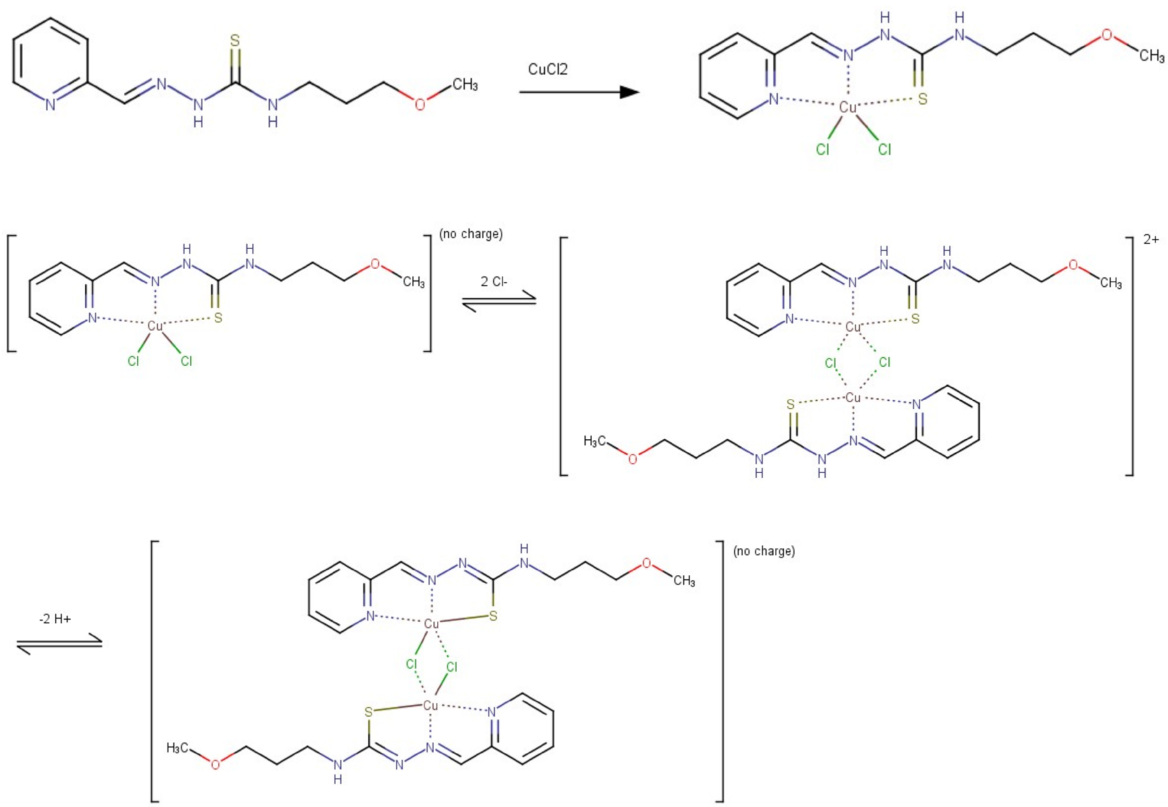

Fragmentation in ESI MS interface

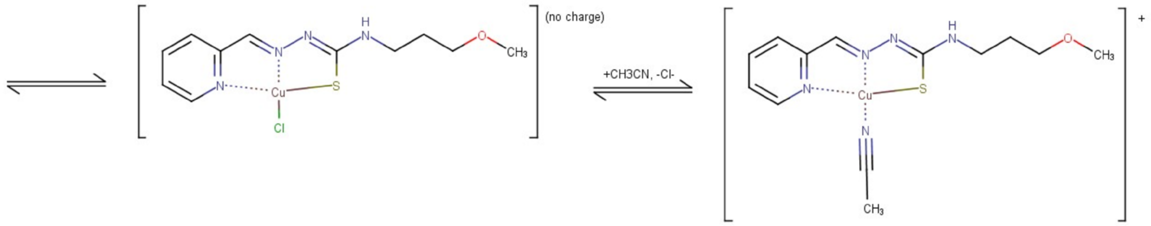

MS only detecting charged species, acetonitrile is the eluent and a likely exchange ligand.
Molecular formula: $C_{13}H_{18}CuN_5OS$, single isotope mass with dominant isotope ^{63}Cu: 355.052806

1.2 Characterisation of VLX50 and VLX60

VLX50

HPLC-UV/MS showed 95.5% purity (254 nm), and $[M + H]^+$ 253.3

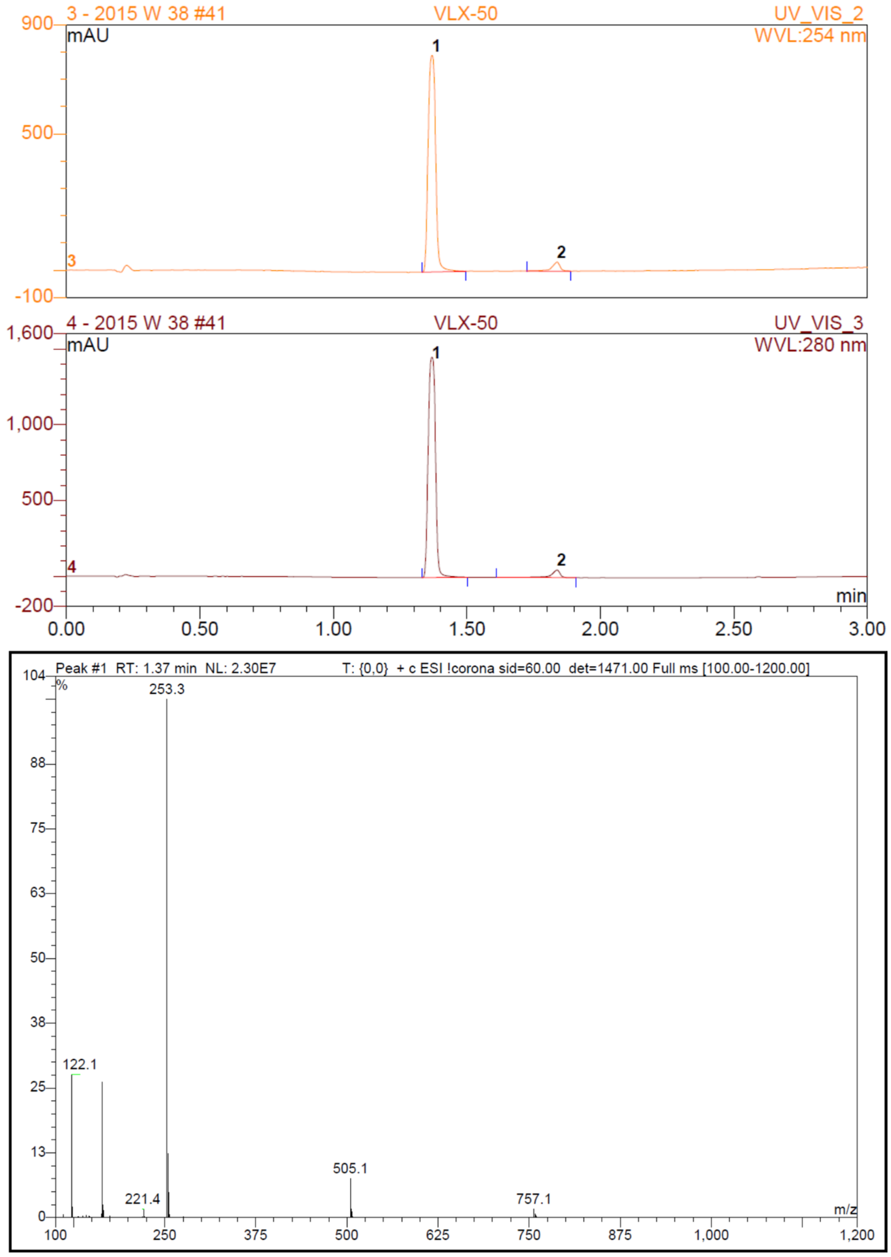

HRMS (ESI): calcd. for $C_{11}H_{16}N_4OS$ $[M + H]^+$ 253.1123; found 253.1120.

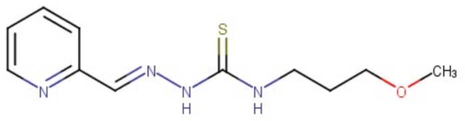

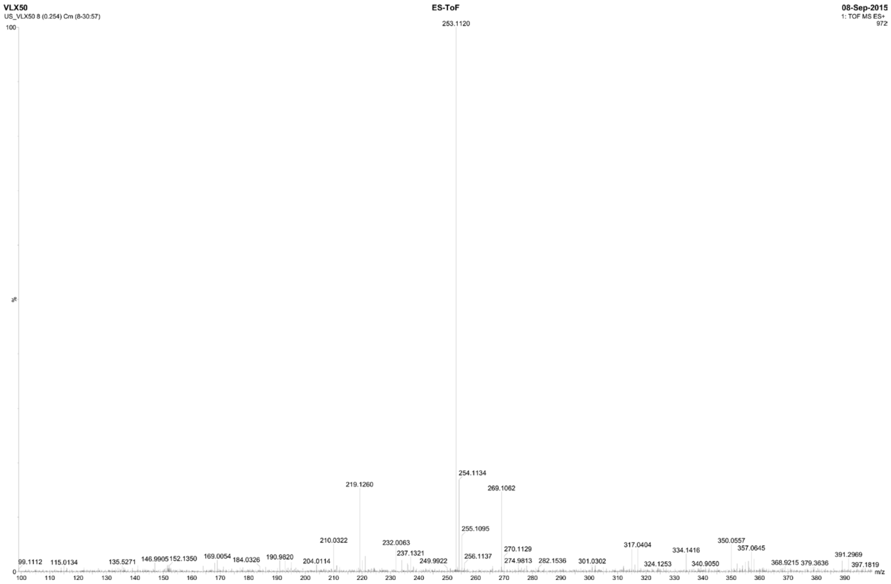

^{1}H NMR (DMSO, 400 MHz): 10.75 (s, 1H), 7.82 (t, 1H, *J* =5.6 Hz), 7.69 (ddd, 1H, *J* = 4.8, 1.7, 1.0 Hz), 7.35 (d, 1H, *J* = 8.1 Hz), 7.23 (s, 1H), 6.99 (td, 1H, *J* = 7.6, 1.4 Hz), 6.52 (ddd, 1H, *J* = 7.3, 5.0, 1.2 Hz), 2.83 (q, 2H, *J* = 6.7 Hz), 2.62 (t, 2H, *J* = 6.0 Hz), 2.48 (s, 3H), 1.73 (quint, 2H, *J* = 1.8 Hz), 1.08 (quint, 2H, *J* = 6.5 Hz).

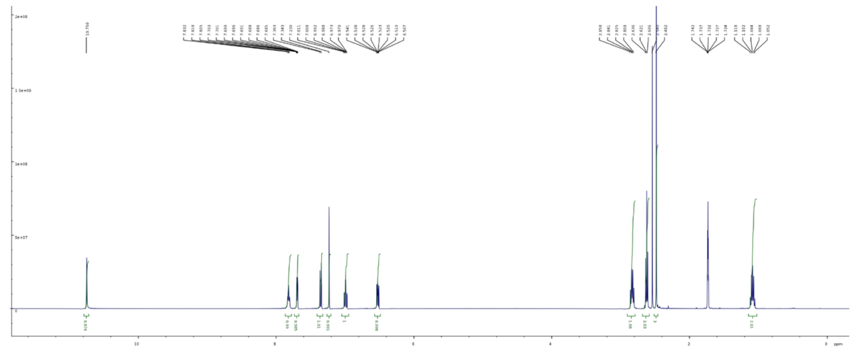

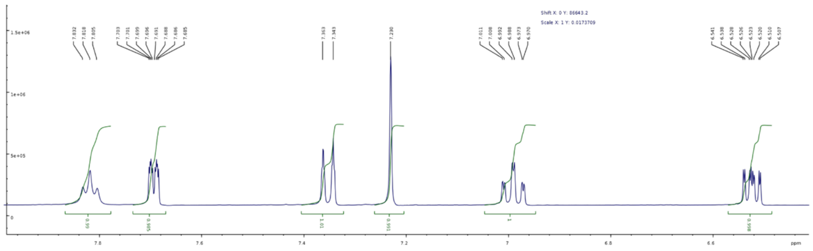

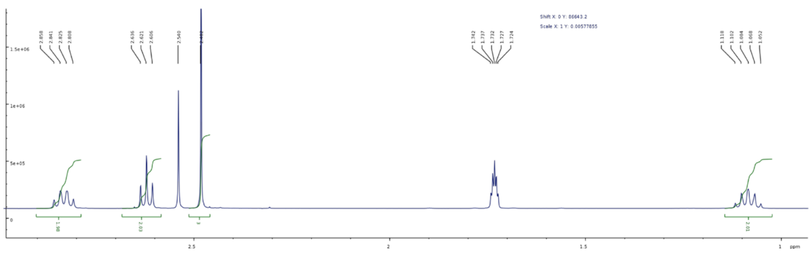

^{13}C NMR (DMSO, 100 MHz): δ 178.1, 154.2, 150.3, 143.0, 137.4, 125.0, 120.9, 71.1, 58.9, 42.5, 29.7

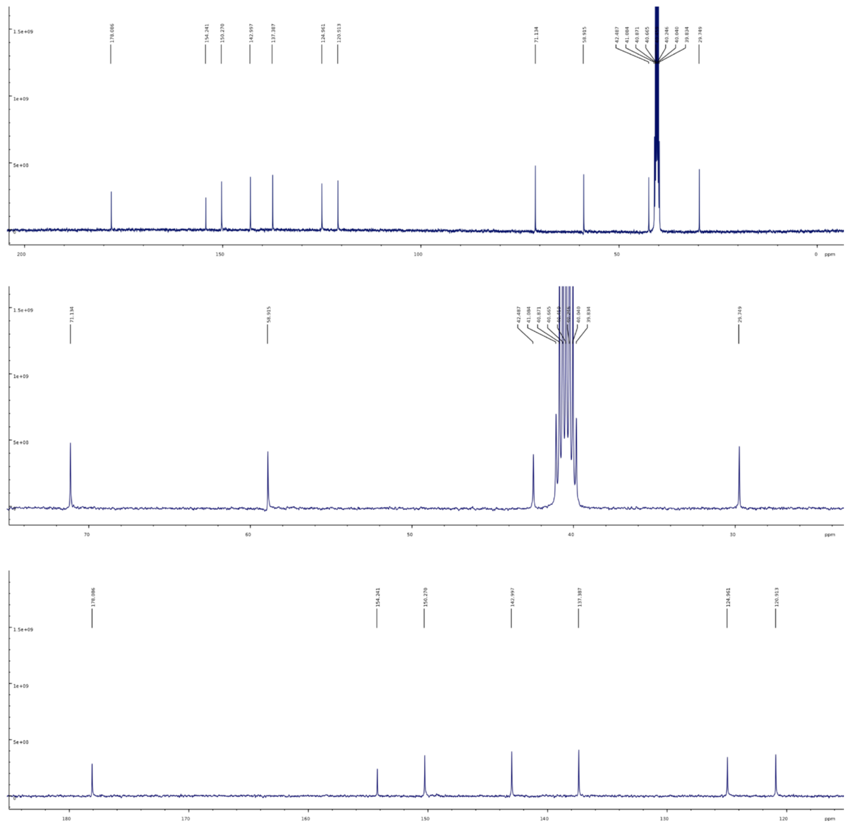

VLX60
95.9% HPLC-UV purity (254 nm)

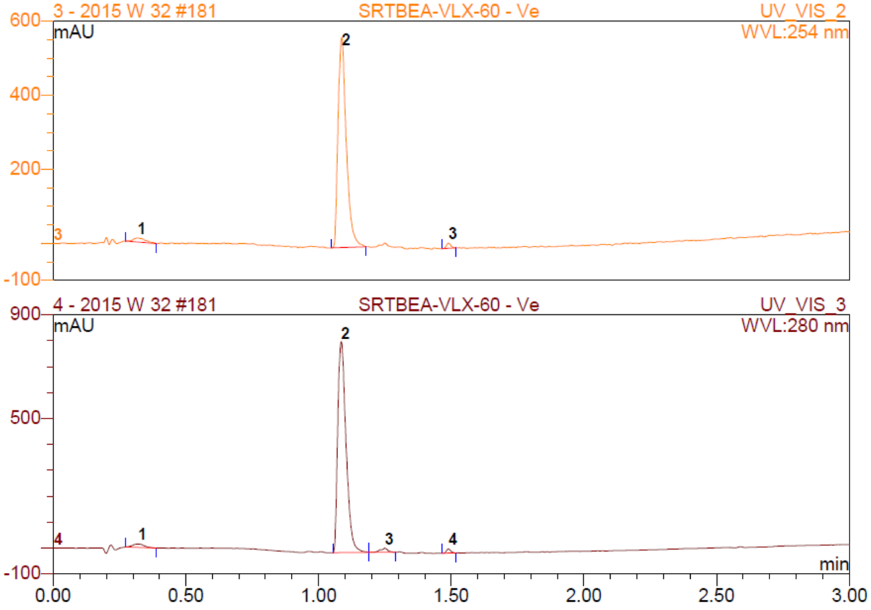

Positive ionization:

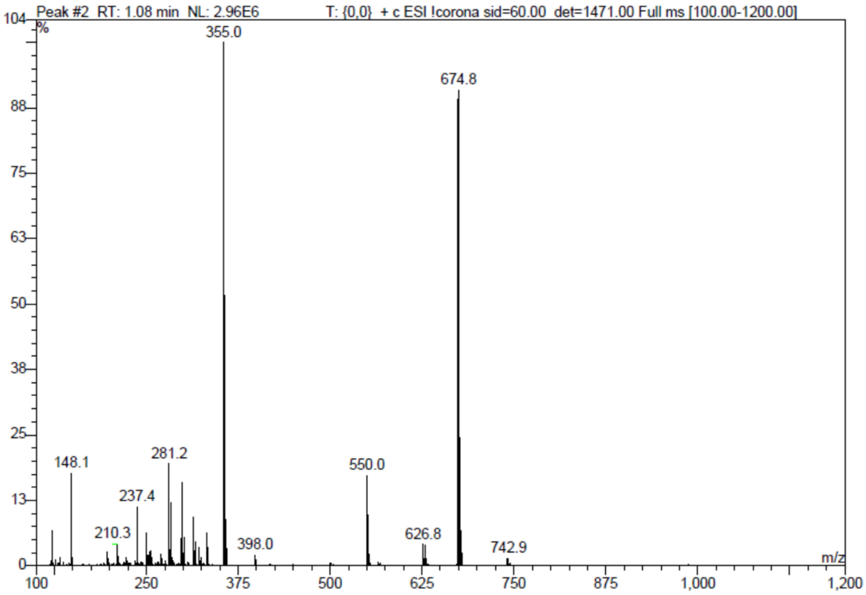

Negative ionization

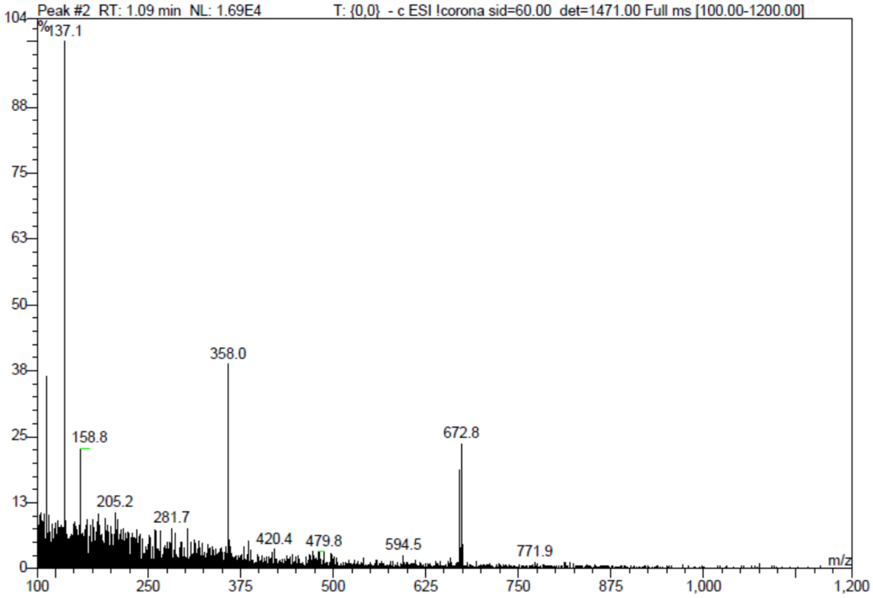

HRMS (ESI): calcd. for C13H18CuN5OS $[M]^+$ 355.052806; found 355.0521 (^{63}Cu) 2.0 ppm dev.
calcd. for C13H18CuN5OS $[M]^+$ 357.050998; found 357.0526 (^{65}Cu); 4.5 ppm dev.

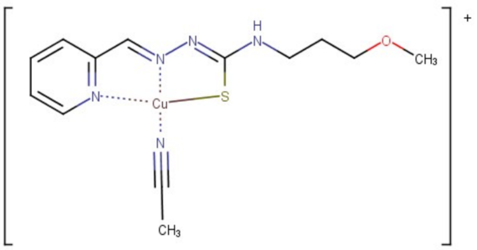

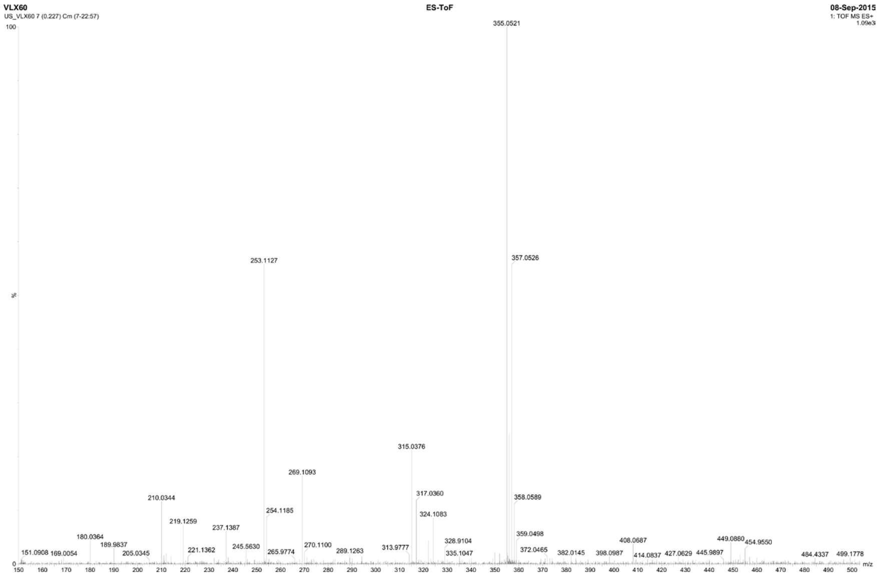

^{1}H NMR (DMSO, 400 MHz): only solvent peaks observed, due to paramagnetic peak broadening of peaks by Cu(II)[2]. Insoluble in $CDCl_3$ and D_2O, partly soluble in DSMO-d_6 and CD_3OD.

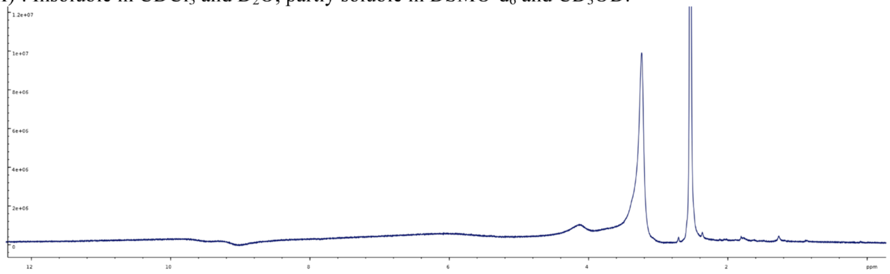

^{13}C NMR (DMSO, 100 MHz): only solvent peaks observed, due to paramagnetic peak broadening of Cu(II). Insoluble in $CDCl_3$ and D_2O, partly soluble in DSMO-d_6 and CD_3OD.

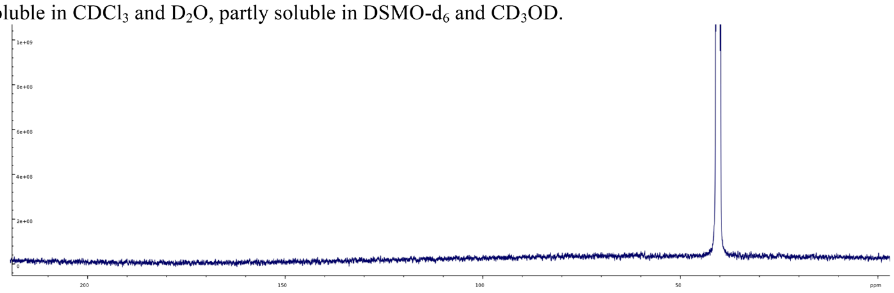

Supplementary Figure 1: Characterization of VLX50 (Maybridge) and VLX60. ^{1}H and ^{13}C NMR spectra were recorded on Varian Mercury Plus instruments: ^{1}H at 399.9 MHz and ^{13}C at 100.6 MHz at 25°C. Chemical shifts (δ) are reported in ppm relative to tetramethylsilane, and spectra were calibrated using residual solvent signals [$(CD_3)_2SO$, ^{1}H at δ = 2.54 ppm, ^{13}C = 40.45 ppm]. Analytical HPLC–UV–MS was carried out with a Dionex UltiMate 3000 HPLC system with a Bruker amaZon SL ion-trap mass spectrometer, and detection by UV (DAD; diode-array detection) and MS (ESI+),using a Phenomenex Kinetex C18 column (50 × 3.0 mm, 2.6 µm particle size, 100 Å pore size) and a flow rate of 1.5 mL/min. A gradient of H_2O/CH_3CN (with 0.05% HCOOH) was used. High-resolution mass spectrometry (HRMS) was carried out with a Micromass Q-Tof2 mass spectrometer equipped with an electro-spray ion source.

2.0 Normal cells compared to tumor cells

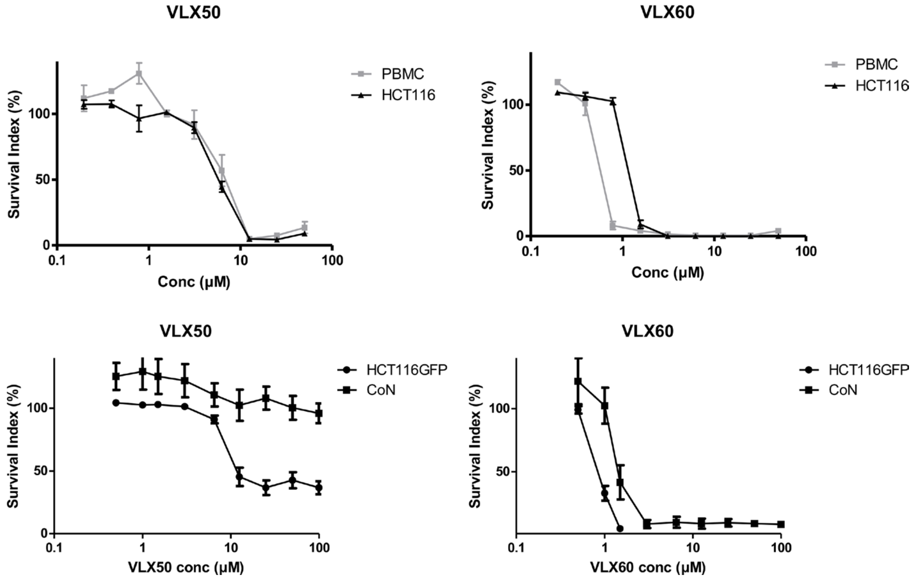

Supplementary Figure 2: Cell survival in the FMCA assay, expressed as survival index, of HCT116, CCD 841 CoN or peripheral blood mononuclear cells (PBMC) cells when exposed to VLX50 or VLX60 for 72 h. Concentration-response curves are based on two (upper panel) to three (lower panel) independent monolayer experiments with triplicate (upper panel) or quadruplicate (lower panel) wells for each concentration. HCT116 and CCD 841 CoN cells are described in “Materials and Methods”, section “Cell lines” and PBMC were obtained from healthy blood donors and prepared as described for leukemic cells in section “Patient tumor cells”. Measurement of cytotoxicity was performed in 96-well plates (upper panel) and 384-well plates (lower panel) as described in section “Measurement of cytotoxicity in monolayer cultured cell lines and patient tumor cells”.

3.0 $CuCl_2$ and $FeCl_3$

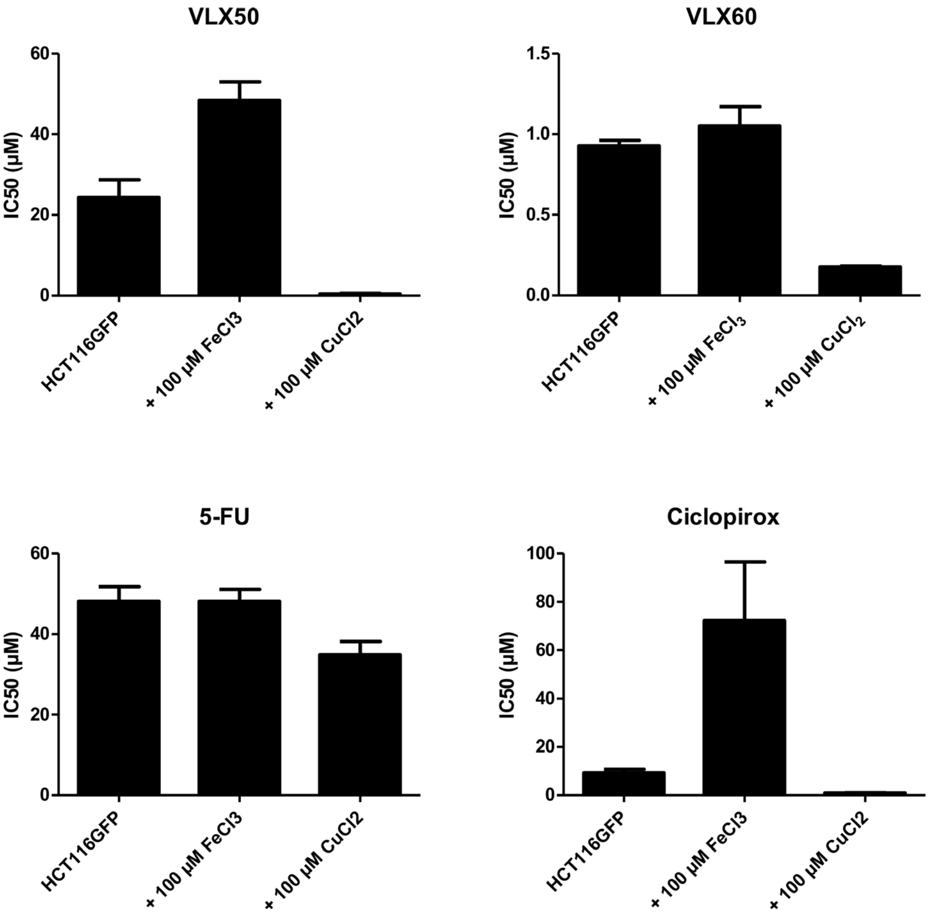

Supplementary Figure 3: Effect of $CuCl_2$ and $FeCl_3$ on the cytotoxic effect of VLX50 and VLX60. Measurement of cytotoxicity was performed in 384-well plates as described in Materials and Methods, section "Measurement of cytotoxicity in monolayer cultured cell lines and patient tumor cells". Using the liquid handling system ECHO® 550, 100 µM $CuCl_2$ or $FeCl_3$ were added 30 min before drug. The addition of $CuCl_2$ significantly increased the cytotoxic effect (IC_{50}) of both VLX50 and VLX60 against HCT116 cells ($P < 0.05$), whereas the addition of $FeCl_3$ impaired the cytotoxic effect (IC_{50}) of VLX50 ($P < 0.05$) but not VLX60 ($P = 0.38$). Of note, 100 µM $CuCl_2$ or $FeCl_3$ alone was non-toxic to HCT116 cells (not shown), and the addition of $CuCl_2$ and $FeCl_3$ resulted in no change in the IC_{50}-value of the standard drug 5-FU. The iron chelator ciclopirox was used as a reference substance. Statistical differences were calculated with unpaired Student´s *t*-test.

4.0 Cell cycle

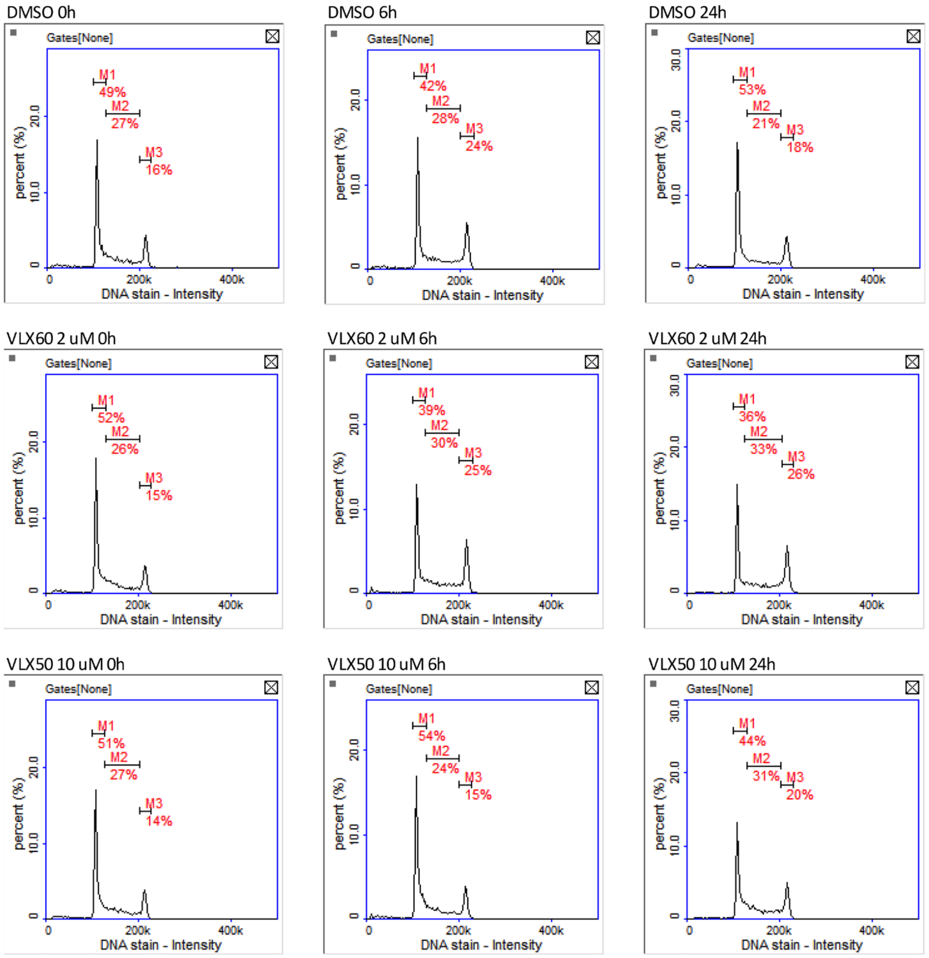

Supplementary Figure 4: Cell cycle analysis. Cell cycle analysis was performed as described in Materials and Methods, section "Cell cycle analysis".

5.0 Gene expression

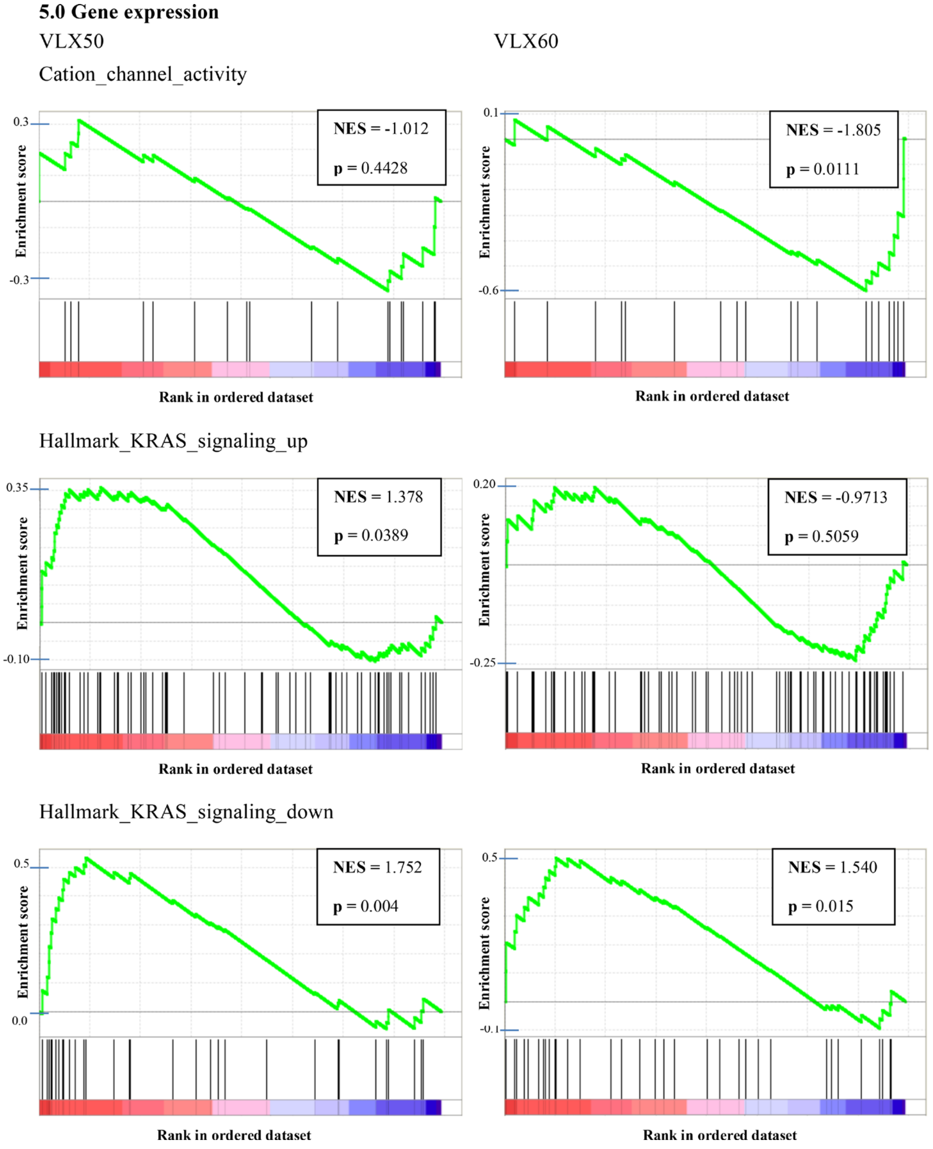

Supplementary Figure 5: Gene set enrichment analysis. Gene expression analysis was performed as described in Materials and Methods, section “VLX 50 and VLX60 mechanistic exploration using gene expression analysis”.

Supplementary Table 1: VLX50_10 uM_6_gene_sets. See Supplementary_Table_1

Supplementary Table 2: VLX60_2 uM_6_gene_sets. See Supplementary_Table_2
